# Ultrafast Formation of Jahn–Teller Polarons Revealed by State-Selective Excitation in Correlated Spinel Co_3_O_4_

**DOI:** 10.1021/jacs.5c23346

**Published:** 2026-04-01

**Authors:** Simone Restelli, Oliviero Cannelli, Nicola Colonna, Carmelo Grova, Paolo Usai, Michele Puppin, Mounir Mensi, Francesco Barantani, Yue Meng, Jérémie Teyssier, Malte Oppermann, Francesco Pennacchio, Camila Bacellar, Jérémy R. Rouxel, Ludmila Diniz Leroy, Oleg Dogadov, Natacha Ohannessian, Daniele Pergolesi, Pietro Galinetto, Przemysław Piekarz, Andrzej Ptok, Swati Chaudhary, Gregory A. Fiete, Majed Chergui, Edoardo Baldini, Giulia F. Mancini

**Affiliations:** † Laboratory for Ultrafast X-ray and Electron Microscopy (LUXEM), Department of Physics, University of Pavia, Pavia 27100, Italy; ‡ Department of Physics, University of Pavia, Pavia 27100, Italy; § Lausanne Centre for Ultrafast Science (LACUS), 27218 École Polytechnique Fédérale de Lausanne, Lausanne 1015, Switzerland; ∥ Laboratory of Ultrafast Spectroscopy (LSU), École Polytechnique Fédérale de Lausanne, Lausanne 1015, Switzerland; ⊥ PSI Center for Scientific Computing, Theory and Data, Paul Scherrer Institute (PSI), Villigen 5232, Switzerland; # Institute of Chemical Sciences and Engineering (ISIC), X-Ray Diffraction and Surface Analytics Platform (XRDSAP), École Polytechnique Fédérale de Lausanne, Sion 1950, Switzerland; ∇ Department of Physics, 12330 The University of Texas at Austin, Austin, Texas 78712, United States; ○ Department of Quantum Matter Physics, University of Geneva, Geneva 1211, Switzerland; ◆ Department of Chemistry, University of Basel, Basel 4056, Switzerland; ¶ SwissFEL, Paul Scherrer Institute (PSI), Villigen 5232, Switzerland; †† Chemical Sciences and Engineering Division, Argonne National Laboratory, Lemont, Illinois 60439, United States; ‡‡ PSI Center for Neutrons and Muons Sciences, Paul Scherrer Institute (PSI), Villigen 5232, PSI Switzerland; §§ PSI Center for Energy and Environmental Sciences, Paul Scherrer Institute (PSI), Villigen 5232, PSI Switzerland; ∥∥ Institute of Nuclear Physics, Polish Academy of Sciences, Kraków 31-342, Poland; ⊥⊥ Department of Physics, 1848 Northeastern University, Boston, Massachusetts 02115, United States; ## Department of Physics, 2167 Massachusetts Institute of Technology, Cambridge, Massachusetts 02139, United States; ∇∇ Elettra-Sincrotrone Trieste S.C.p.A, S.S. 14 km 163,5 in Area Science Park, Basovizza, Trieste 34012, Italy

## Abstract

Jahn–Teller polarons are quasiparticles that stem from symmetry breaking and strong local electron–phonon coupling. They originate from an excess charge carrier being dressed by a local lattice distortion, caused by the Jahn–Teller effect, and they critically impact electrical, structural, and magnetic properties in transition metal oxides. The observation of the microscopic steps involved in their formation is essential for enabling control over material properties through the targeted activation of local, site-specific modifications with light pulses. While Jahn–Teller distortion associated with polaron formation was predicted to contribute significantly to changes in electronic band gap and optical properties in Co_3_O_4_, its experimental observation remains elusive, requiring signatures of local symmetry reduction. In this work, we demonstrate Jahn–Teller polaron formation in spinel Co_3_O_4_. By exciting electronic transitions at 3.10 eV and 1.55 eV, we target either the *O*_*h*_ Cobalt­(III) or the *T*_*d*_ Cobalt­(II) ions, and drive the subsequent coherent responses of the system through two different pathways. For the former, we demonstrate that ligand-to-metal charge transfer leads to Jahn–Teller polaron formation, which is linked to the deformation potential and magnetoelastic coupling. For the latter, we identify the coherent excitation of a T_*2g*_ phonon mode launched by on-site d-d electronic transitions. Key to our observations is the ability to target site-specific electronic excitations in spinel Co_3_O_4_ using ultrafast optical pulses, while monitoring the ensuing low-energy collective modes through the coherent time-domain response of the material. Our approach, which combines the comparative analysis of experimental fingerprints with the support from density functional theory calculations, is broadly applicable to systems in which Jahn–Teller polaron physics has been theoretically predicted but remains experimentally unverified, and it underscores the potential of electronic-state targeting as a route to selectively excite and probe quasiparticle dynamics in solids.

## Graphical abstract

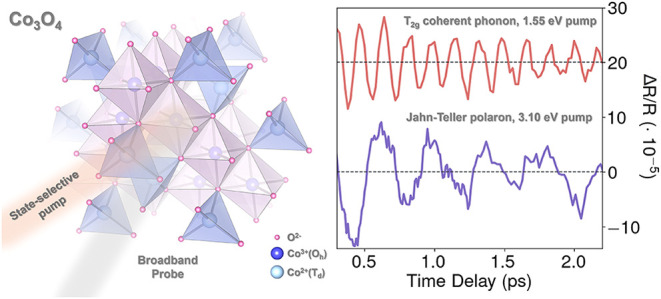

## Introduction

Jahn–Teller polarons are quasiparticles caused by an excess charge carrier being dressed by a lattice distortion through electron–phonon coupling.^1^ They stem from local symmetry-breaking caused by the Jahn–Teller effect,^2,3^ which uplifts inherent electronic instability caused by the presence of degenerate electronic states. Their origin is fundamentally different from other types of polarons, i.e., Fröhlich polarons, where the coupling of a charge carrier occurs with existing, long-range, lattice distortions which preserve the lattice symmetry even when nontotally symmetric phonons are activated.^4,5^

Transition metal oxides (TMOs) are heavily affected by Jahn–Teller effects. They feature coupled charge, orbital, magnetic, and lattice degrees of freedom, placing their ground state on the cusp of multiple phases due to a delicate balance of different microscopic interactions.^6^ These systems, which include unconventional superconductors,^7^ charge- and orbitally ordered systems,^8,9^ and quantum magnets,^10^ often exhibit exotic phenomena emerging from intricate interactions that challenge our understanding of matter.^11^

Jahn–Teller polarons provide an effective mechanism for tuning the energies of electronic transitions in TMOs, thereby influencing key properties such as electrical conductivity, optical response, and colossal magnetoresistance.^12,13^ In manganites, they can enhance resistivity by trapping charge carriers, leading to activated hopping conduction.^14,15^ In cuprates, Jahn–Teller effects have been conjectured to be linked to superconductivity and charge ordering.^16,17^ Moreover, Jahn–Teller polarons can also be affected by magnetic and spin–orbit interactions, leading to more-complex quasiparticles such as spin–orbital polarons, which can exhibit unique properties and behaviors,^18^ e.g., preserving the Mott gap upon doping 5d TMOs, thereby preventing the transition to a metallic phase.^19^

The observation of the microscopic steps through which Jahn–Teller polaron formation occurs in TMOs critically requires controlling their electronic properties with light pulses through the activation of local and site-specific lattice distortions. Moreover, it requires transient signatures of local symmetry reduction combined with their targeted activation.

Spinel Cobalt Oxide (Co_3_O_4_) is a correlated TMO characterized by the copresence of tetrahedral high-spin Cobalt­(II) and octahedral low-spin Cobalt­(III) sites. This system hosts Mott-Hubbard, charge transfer, and on-site d-d excitations in the energy range of near-infrared and visible light,^20^ resulting in a rich optical absorption spectrum. Since its absorption bands favorably overlap the solar spectrum, spinel Co_3_O_4_ has been considered a candidate for all-TMO solar cells,^21^ possibly complementing lead-halide perovskite based solar cells, in which Fröhlich polarons were suggested to affect charge carriers stabilization and transport.^5^ The presence of Co­(III) in edge-sharing neighboring octahedral sites was shown to influence Co_3_O_4_ charge transport and surface reactivity via a nearest-neighbor hopping conduction mechanism, affecting its catalytic and conductivity properties.^22^

Local polarons associated with Jahn–Teller distortions were predicted in Co_3_O_4_ by first-principles calculations of the optical absorption, arguing a stronger contribution from the *d* states of the Co­(III) octahedral centers to the calculated density of states, with respect to the Co­(II) tetrahedral sites.^23^ However, Jahn–Teller polaron formation has not been unambiguously identified because the coexistence of metal ions in different charge and spin configurations, along with lattice sites of different symmetry, complicates the identification of site-selective activation protocols in Co_3_O_4_. Moreover, observing Jahn–Teller polaron formation requires an intrinsically time-resolved approach, as the process is inherently transient.

To address this challenge, we perform state-selective broadband transient reflectivity and monitor the low-energy collective modes of Co_3_O_4_ through its coherent response in the time domain (Figure 1). We demonstrate the possibility to target site-specific excitations with optical light pulses, guiding the system through two different relaxation pathways. Upon 3.10 eV photoexcitation, the selective ligand-to-metal charge transfer process between O^2–^ and Co^3+^ octahedral sites leads to local symmetry breaking and Jahn–Teller polaron formation, which influences the deformation potential and magnetoelastic coupling of the system. This scenario is supported by density functional theory (DFT) calculations, which demonstrate the formation of stable polaronic in-gap states, with local symmetry breaking associated with charge localization on the 3d_*z* ^2^_ orbital at the Co­(*O_h_*) site. To our knowledge, this is the first time this type of physics is unraveled with this microscopic detail in this material. When we excite at a laser pump energy of 1.55 eV, the system follows a completely different mechanism: the selective modulation of the Co^2+^ *T*_*d*_ sites of Co_3_O_4_ leads to the coherent excitation of a T_*2g*_ phonon mode launched by on-site d-d electronic transitions, through electron–lattice coupling.

**Figure 1:**
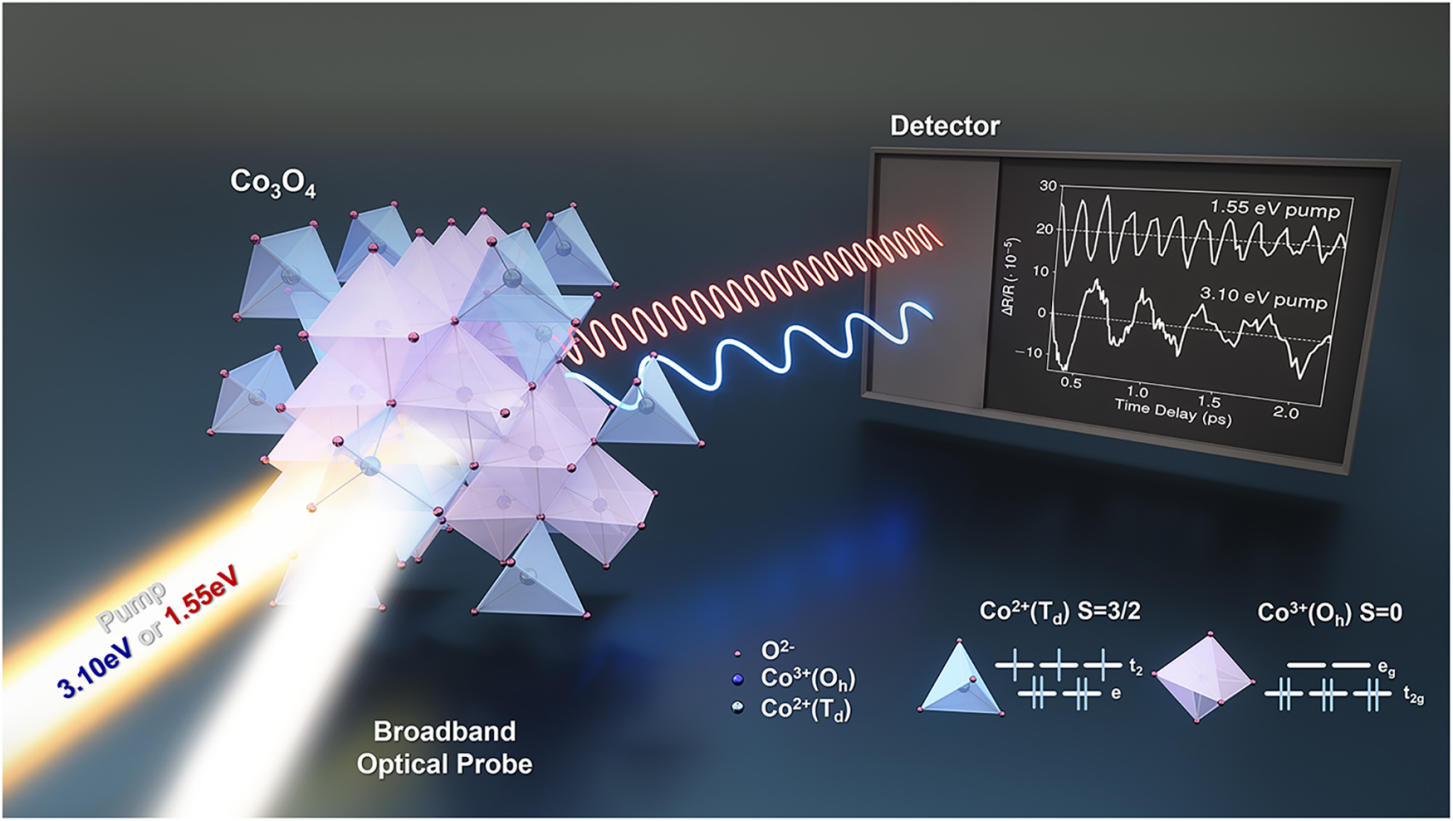
Schematic layout of the state-selective photodoping experiment in the correlated spinel Co_3_O_4_. Transient reflectivity measurements used 1.55 eV and 3.10 eV pump pulses and a broadband optical probe, revealing different responses in the sample and, for the latter, Jahn–Teller polaron formation. Inset: cobalt tetrahedral and octahedral local geometries, electronic configurations, and spin states in spinel Co_3_O_4_. An arbitrary offset and intensity scaling was introduced in the traces at the detector for clarity. Sample and technical measurement details are provided in the SI. Courtesy of Balázs Őrley.

Our methodological approach provides a viable framework for interpreting Jahn–Teller polaron physics in transition-metal oxides, as well as in other materials whose structural or electronic complexity renders first-principles modeling computationally intensive.

### Optical Transitions and Phonon Modes in Co_3_O_4_

Spinel Co_3_O_4_ is a correlated insulator with the *O*_*h*_^7^ – *Fd*3̅*m* space group at room temperature (RT). It features Co^2+^ and Co^3+^ centers in a 1:2 stoichiometric ratio that occupy tetrahedral and octahedral sites with O^2–^ vertices, respectively (Figure 1). The crystal-field splitting produces a high-spin configuration for the Co^2+^(d^7^) sites (*S* = 3/2), which form ionic bonds with the neighboring O^2–^ ions, whereas the octahedral symmetry leads to a low-spin state for the Co^3+^(d^6^) sites (*S* = 0), characterized by a strong hybridization with the ligands.^24^ The optical conductivity spectrum of Co_3_O_4_, obtained from ellipsometry measurements, shows four optical transitions in the 1.40–4.30 eV energy range (Figure 2a), which are assigned to^20,24^ Mott–Hubbard (MH) transition from Co^3+^ to Co^2+^ (feature A); on-site d-d excitations within Co^2+^ and Co^3+^ (feature B); ligand-to-metal charge transfer (LMCT) transition between O^2–^ and Co^2+^ (feature C) and Co^3+^ (feature D). Below the Néel temperature *T*_N_ = 30–40 K, the system retains the cubic lattice symmetry^25^ while undergoing an antiferromagnetic (AFM) transition. The low-temperature magnetic phase is characterized by Co^2+^ sites forming two face-centered-cubic sublattices with opposite magnetic moments coupled via superexchange interactions, while Co^3+^ centers retain *S* = 0.^26^ The system hosts five Raman-active phonon modes, as observed in the spontaneous Raman spectra collected for excitation energies tuned to three different electronic transitions (Figure 2b): on-site d-d transitions of the Co centers at 1.57 eV (red trace), low energy side of the O^2–^ to Co^2+^ charge transfer (CT) transition at 2.33 eV (gray trace), and LMCT transitions at 3.06 eV (blue trace). Based on our DFT calculations, which show excellent agreement with the experiment (gray and red diamonds at the Γ point in Figure 2c and Table S2 in the Supporting Information, SI), we assign these features to the following phonon modes: 24.3 meV (T_*2g*_), 59.9 meV (E_*g*_), 64.8 meV (T_*2g*_), 77.1 meV (T_*2g*_), and 85.9 meV (A_*1g*_). These results are fully consistent with the literature^27^ (Table S2).

**Figure 2:**
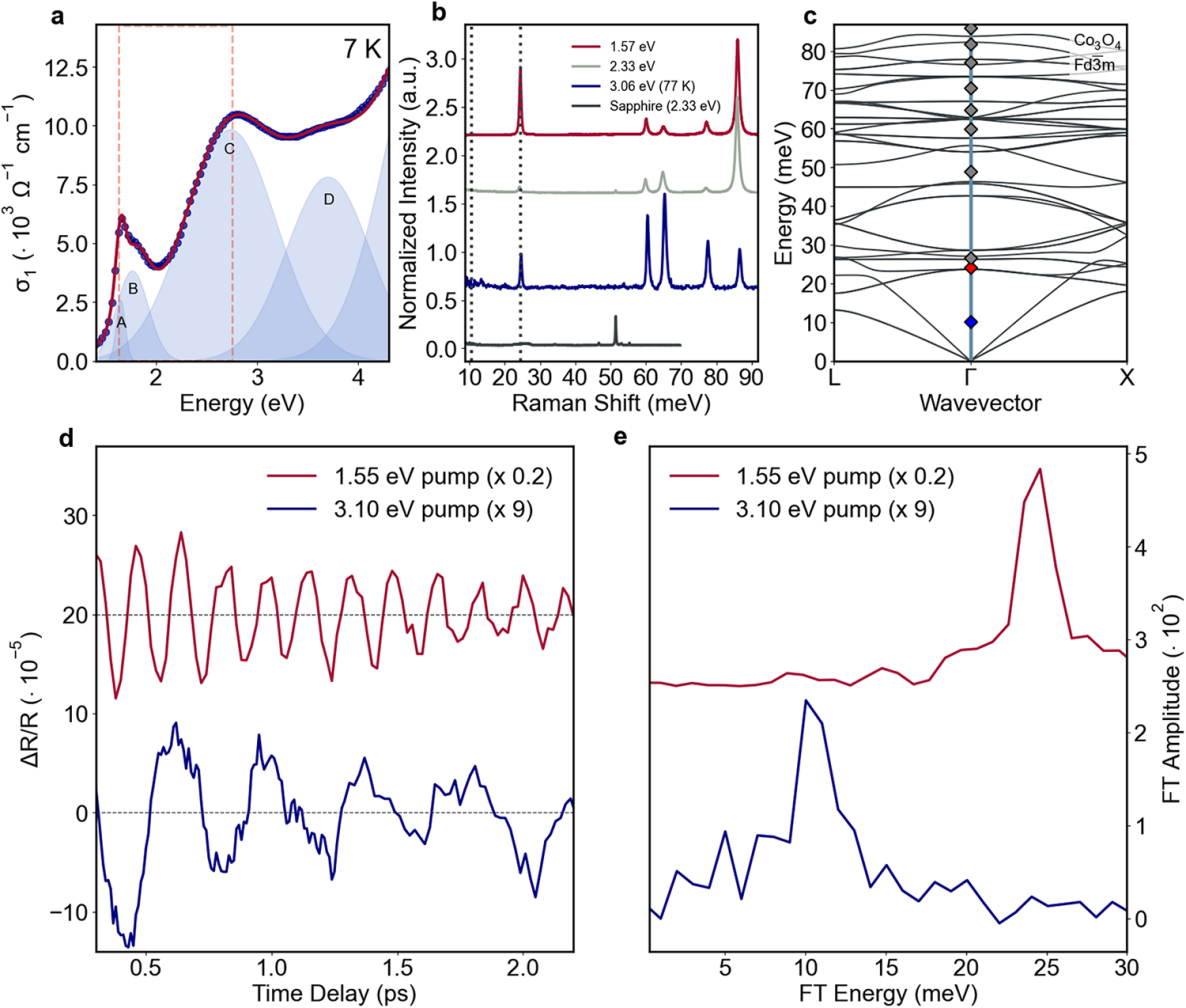
Main results of state-selective excitation in Co_3_O_4_. (a) Co_3_O_4_ optical conductivity (σ_1_) extracted from visible-ultraviolet ellipsometry measurements performed at 7 K in the 1.40–4.30 eV energy region (blue dots) and its fit (red solid line) with Gaussian functions (light blue). The relevant spectral components are labeled from A to D. The dashed orange rectangle highlights the probe energy region of the transient reflectivity measurements. (b) Spontaneous resonant Raman spectra collected upon: 1.57 eV excitation at RT (red); 2.33 eV excitation at RT (gray); 3.06 eV excitation at 77 K (blue). The slight hardening of the 3.06 eV spectrum is due to its lower acquisition temperature (77 K) compared to the other two spectra, measured at RT. The black trace corresponds to the Raman spectrum of the sapphire substrate background (see details in the SI) upon 2.33 eV excitation at RT. The dashed vertical lines mark the energy of the coherent oscillations observed in the transient reflectivity experiment. (c) DFT phonon dispersion along the Γ–*L* and Γ–*X* high symmetry directions. The gray diamonds correspond to the phonon energies measured with steady-state experiments; the red and blue diamonds mark the energies measured in the time-resolved experiments upon 1.55 eV and 3.10 eV excitation, respectively. (d) Coherent oscillatory response of Co_3_O_4_ upon 1.55 eV excitation with 1.4·10^20^ cm^–3^ photocarrier density (red) and 3.10 eV excitation with 4.9·10^20^ cm^–3^ photocarrier density (blue). The measurements were taken at 70 K (above *T*_*N*_) in the 1.70–1.80 eV probe region. (e) FT of the Co_3_O_4_ coherent responses upon 1.55 eV (red) and 3.10 eV excitation (blue), respectively peaked at 24.3 ± 2.0 meV and 10.2 ± 1.5 meV. The Co_3_O_4_ spectra in (b) were normalized by the mode of maximum intensity. An arbitrary offset was introduced in panels (b, d and e) for clarity.

### Pump–Probe Reflectivity Measurements

To unravel how different low-energy modes dynamically couple to high-energy transitions in Co_3_O_4_, we perform broadband transient reflectivity. We investigate the material’s out-of-equilibrium response upon state-selective electronic excitations following the experimental methodology detailed in Section S2 of the SI. Figure 2d shows the transient reflectivity coherent responses of Co_3_O_4_ above *T*_*N*_, integrated over the 1.70–1.80 eV energy range. These are selectively triggered upon on-site d-d (1.55 eV pump) and LMCT (3.10 eV pump) excitations. We observe oscillation periods of 167 ± 20 fs (red trace) and 405 ± 20 fs (blue trace) that persist beyond 2 ps. Figure 2e shows their Fourier transform (FT) analysis. The spectra are characterized by a peak at 24.3 ± 2.0 meV for the 1.55 eV excitation (red trace) and a feature at 10.2 ± 1.5 meV for the 3.10 eV excitation (blue trace). The amplitude of both modes scales linearly with the laser intensity and therefore with the photocarrier density (Figures S23 and S25 of the SI). While the former mode energy has an excellent agreement with that of the lowest T_*2g*_ mode of the *Fd*3̅*m* structure (Figure 2b), and with the DFT phonon dispersion at the Γ point (red diamond in Figure 2c), the latter is atypical. Indeed, it is not present in the steady-state Raman spectra at any resonant excitation energy, nor in the phonon structure at the Γ point (blue diamond in Figure 2c), suggesting that this mode does not belong to the ground state structure.

#### On-Site d-d Selective Response

To gain insights into these results, we first study the amplitude of the coherent response for the 1.55 eV excitation (resonant with feature B, Figure 2a) as a function of the FT energy and probe photon energy (Figure 3a). The 24.3 meV feature of the T_*2g*_ phonon is present in the 1.70–1.80 eV probe photon energy region and peaks around 1.70 eV. We fit the oscillatory response with a damped cosine-like function (Figure S22 of the SI), and obtain a damping time constant *t*_D_ = 4.2 ± 2.0 ps and a phase φ = 0.02 ± 0.08 rad, which indicates a displacively excited phonon.^28–30^ This assignment agrees with the linear scaling of the FT amplitude with the excitation density (Figure 3b, pink squares), and with the independence of the FT energy of the coherent oscillation with the temperature (Figure 3b, blue stars). Moreover, our DFT calculations show that the eigenvector of the 24.3 meV mode involves a lattice displacement of the Co^2+^ and O^2–^ ions, while the position of the Co^3+^ centers remains unchanged (Figure 3c). As such, the 1.55 eV excitation instantaneously modifies the charge distribution in the material, shifting the equilibrium position of the Co–O bonds and generating a restoring force that initiates the T_*2g*_ phonon motion. The presence of a selective structural response around the tetrahedral sites provides evidence for the assignment of the on-site d-d transition to the Co^2+^ centers only, rather than both Co^2+^ and Co^3+^ sites.^20,24^ Therefore, the 1.55 eV excitation leads to a selective modulation of mainly the *T*_*d*_ sites of Co_3_O_4_.

**Figure 3:**
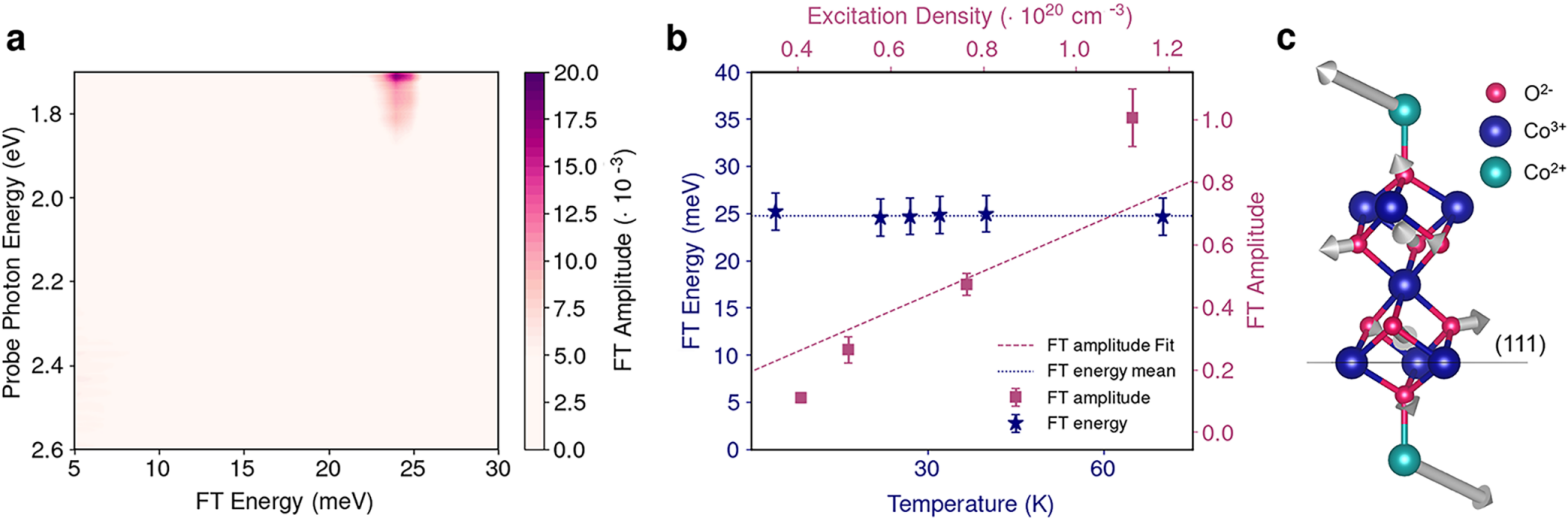
Coherent T_*2g*_ phonon mode observed in correlated spinel Co_3_O_4_ upon selective on-site d-d excitation at 1.55 eV. (a) FT amplitude as a function of the FT energy and probe energy upon 1.55 eV excitation at 70 K. The excitation is resonant with the on-site d-d excitation (feature B, Figure 2a) (b) FT energy of the coherent oscillation (blue stars, left axis) as a function of temperature (bottom axis) with its mean value (dotted line), and FT amplitude (pink squares, right axis) as a function of the excitation density (top axis) with its linear fit (dashed line). The observed behavior corroborates the assignment to a coherently excited phonon. (c) Rendering of the Co_3_O_4_ *Fd*3̅*m* primitive cell comprising octahedral Co^3+^ sites (blue), tetrahedral Co^2+^ sites (turquoise), and O^2–^ (pink) sites. The solid black line shows the (111) sample plane. Pump pulses at 1.55 eV selectively activate the 24.3 meV phonon at the Γ point, characterized by the distortion indicated by the gray arrows. This coherent phonon is a mode also present in the ground state of the system (Figure 2b).

#### Jahn–Teller Polaron Selective Response

The physical origin of the coherent oscillation emerging upon 3.10 eV photoexcitation is less straightforward to assign. Here, we argue that this mode is associated with the formation of a Jahn–Teller (JT) polaron.

State-selective excitation at 3.10 eV induces a LMCT transition from the O^2–^ to Co^3+^ ions,^31,32^ causing an oxidation state change of the Co^3+^ centers to Co^2+^(pump resonant with feature D, Figure 2a), while preserving their octahedral coordination. Since these sites are strongly JT active, due to the degeneracy of the t_*2g*_^6^e_*g*_^1^ electronic configuration, the out-of-equilibrium local structure must have a displaced minimum energy geometry with respect to the ground state.^33,34^ As a consequence, the ultrafast generation of Co^2+^ (*O*_*h*_) sites triggers the activation of a coherent vibration around a new equilibrium position, which is detected in transient reflectivity as a periodic modulation of the signal.

JT local symmetry breaking is directly linked to the deformation potential, which describes how the energy levels of electrons in a material change upon interaction between excess carriers and lattice vibrations that arise during deformation. Due to the peculiar spin configuration of spinel Co_3_O_4_, this process involves magnetoelastic coupling, mediated by spin interactions.

Our experimental observables, summarized in Figure 4, show a modulation of the MH excitation from the Co^3+^ to the Co^2+^ (feature A, Figure 2a) across the AFM transition as a consequence of the JT polaron formation. The photoinduced JT effect reflects in a change of the electronic transition energies with the interplay of the spin degrees of freedom. Figure 4a,b show the FT amplitude and phase maps as a function of the FT energy and probe photon energy in the 1.65–1.77 eV region for two selected temperatures above (70 K, panel a) and below (4.2 K, panel b) the Néel temperature. At 70 K, we observe a single peak at 10.2 meV with constant zero radiant phase across the entire probe energy region. Instead, at 4.2 K the peak splits in two features lying in the ranges 1.65–1.70 eV, located around the low-energy inflection point of the on-site d-d excitation (feature B in Figure 2a), and 1.72–1.76 eV, centered on the high-energy inflection point of the MH transition (feature A in Figure 2a). Figure 4b shows that these two regions are in opposite phase to each other, reflecting a modulation of the underlying transition’s energy caused by the oscillating mode.^35–38^ We note that the assignment of the 10.2 meV collective excitation to a coherent optical phonon agrees with the linear scaling of the oscillation amplitude with the excitation density, and with the independence of the mode energy with respect to the temperature and the probe photon energy (Figure 4c and Section S5.3.3 of the SI).

**Figure 4:**
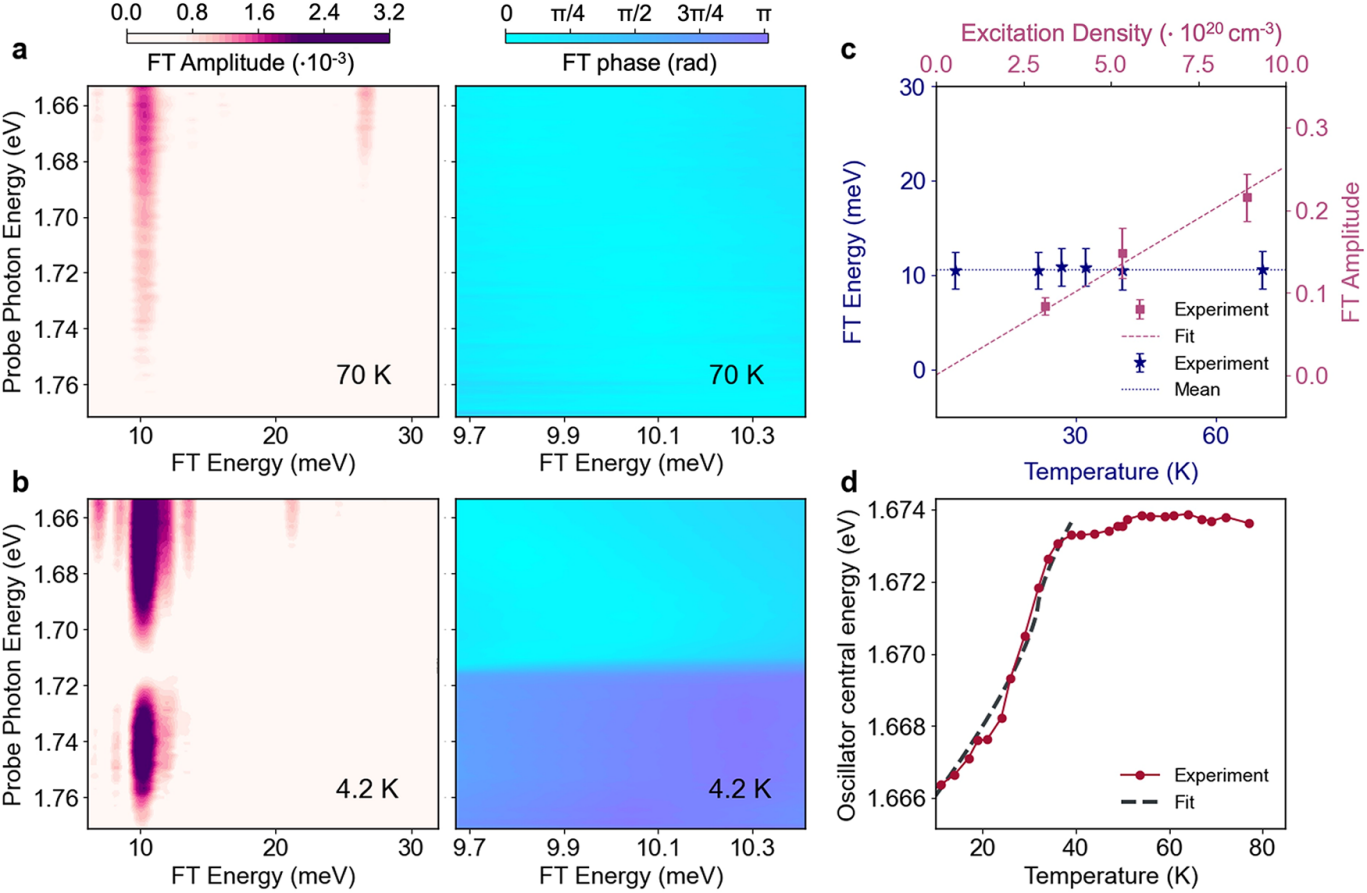
Jahn–Teller polaron formation observed in correlated spinel Co_3_O_4_ upon selective charge-transfer excitation at 3.10 eV. (a, b) FT amplitude (left) and phase (right) as a function of the FT frequency and probe energy upon 3.10 eV excitation and with 4.9·10^20^ cm^–3^ photocarrier density above (70 K, panel a) and below *T*_*N*_ (4.2 K, panel b). Phase and amplitude panels share the same probe photon energy axis. (c) FT energy of the coherent oscillation (blue stars, left axis) as a function of temperature (bottom axis) with its mean value (dotted line), and FT amplitude (pink squares, right axis) as a function of the excitation density (top axis) with its linear fit (dashed line). (d) Ellipsometry measurement of the MH transition from the Co^3+^ to the Co^2+^ energy as a function of temperature. The 3.10 eV pump induces a Jahn–Teller symmetry breaking that triggers the 10.2 meV coherent mode. This, in turn, influences the MH central energy across the AFM transition through the magnetoelastic effect described by our model (dashed line).

The ellipsometry data in Figure 4d show how the energy of the MH transition from the Co^3+^ to the Co^2+^ (feature A) evolves across the Néel temperature. We observe a plateau in the 40–75 K range, followed by an 8 meV redshift from 40 to 7 K. According to the theoretical description developed by Callen,^39^ we ascribe this change to a magnetoelastic coupling. In magnetic insulators, the energy of an interband transition depends on the spin correlation between two nearest neighbor sites ⟨*S*_*i*_ ·*S*_*j*_⟩ as:

1 $$E\propto \sum_{i,j}D_{\mathrm{ij}}\left\langle S_{i}\cdot S_{j}\right\rangle$$

The proportionality constant contains a term accounting for the deformation potential,^40^ and the sum runs over all pairs of nearest neighbor sites. *D*_*ij*_ is the derivative of the *J*_*ij*_ exchange constant with respect to the volume strain Δ*V*: $D_{\mathrm{ij}}=\frac{dJ_{\mathrm{ij}}}{d\Delta V}$ . We can evaluate the spin correlation function as:

2 $$\left\langle S_{i}\cdot S_{j}\right\rangle ;\propto m{\left(T\right)}^{2}\propto {\left(T-T_{N}\right)}^{2\beta }$$

where *m*(*T*) is the reduced magnetization and β is the critical exponent of the order parameter. We fit the MH transition energy as a function of the temperature with this model (Section S4 of the SI), obtaining *T*_*N*_ = 32 K, which is consistent with previous studies.^25,26^

The agreement between the fit and our experimental ellipsometry data (Figure 4d) provides evidence of the dependence of the MH excitation from the Co^3+^ to the Co^2+^ (feature A) energy on structural changes, through the mediation of spin interactions. The activation of nuclear displacements, by changing the unit cell volume, acts on the deformation potential and on the *D*_*ij*_ terms. Ultimately, this induces an oscillatory modulation of the electronic transition energy over time that leads to the phase change reported in Figure 4b. By performing similar fits, we analyzed the magnitude of the magnetoelastic coupling on the higher energy excitations (Figure S17 of the SI). We found that a similar effect could also be present for the 2.70 eV transition (LMCT between O^2–^ and Co^2+^), which, however, has an inflection point outside the probe energy range of our experiment. The above results establish a connection between the electronic response and the deformation potential, mediated by spin interactions, thereby suggesting a structural origin of the 10.2 meV mode via symmetry breaking due to the formation of a JT polaron.

To corroborate the proposed scenario, we simulate Jahn–Teller polaron formation in spinel Co_3_O_4_ through a constrained DFT + *U* calculation in Quantum ESPRESSO^41^ on a 2 × 2 × 2 supercell (Section 2.3 of the SI). LMCT-driven oxidation state change of the Co^3+^(*O_h_*) to Co^2+^(*O_h_*) is simulated by introducing an excess −1 charge localized on the octahedral Cobalt site, and constraining the total spin of the system to +1. The electronic density of states (DOS) changes from the pristine (Figure 5a) to the polaronic structure (Figure 5b), showing the appearance of localized stable in-gap states, characterized by a dominant contribution of the Co­(*O_h_*) center (Figure 5b, purple trace). The lattice distortion accompanying the appearance of these states, due to JT polaron formation, is reported in Figure 5c, and it results from tracking the changes in atomic positions during the relaxation of the supercell toward the polaronic energy minimum. The structural changes are asymmetric, with a dominant distortion along the crystallographic *a*-axis, which causes a local breaking of the cubic symmetry. As the elongation of the apical oxygens removes the e_*g*_ orbital degeneracy and lowers the energy of the 3d_*z* ^2^_ orbital, the excess charge is localized on the 3d_*z* ^2^_ orbital of a Co­(*O_h_*), a finding that is demonstrated by our calculation (Figure 5d). Our results, therefore, show that the JT polaron is stable and it arises in connection to a local symmetry breaking around the photodoped Co­(*O_h_*) site, of which we detail the microscopic description, with the overall supercell structural change reported in the Section 2.3 of the SI.

**Figure 5:**
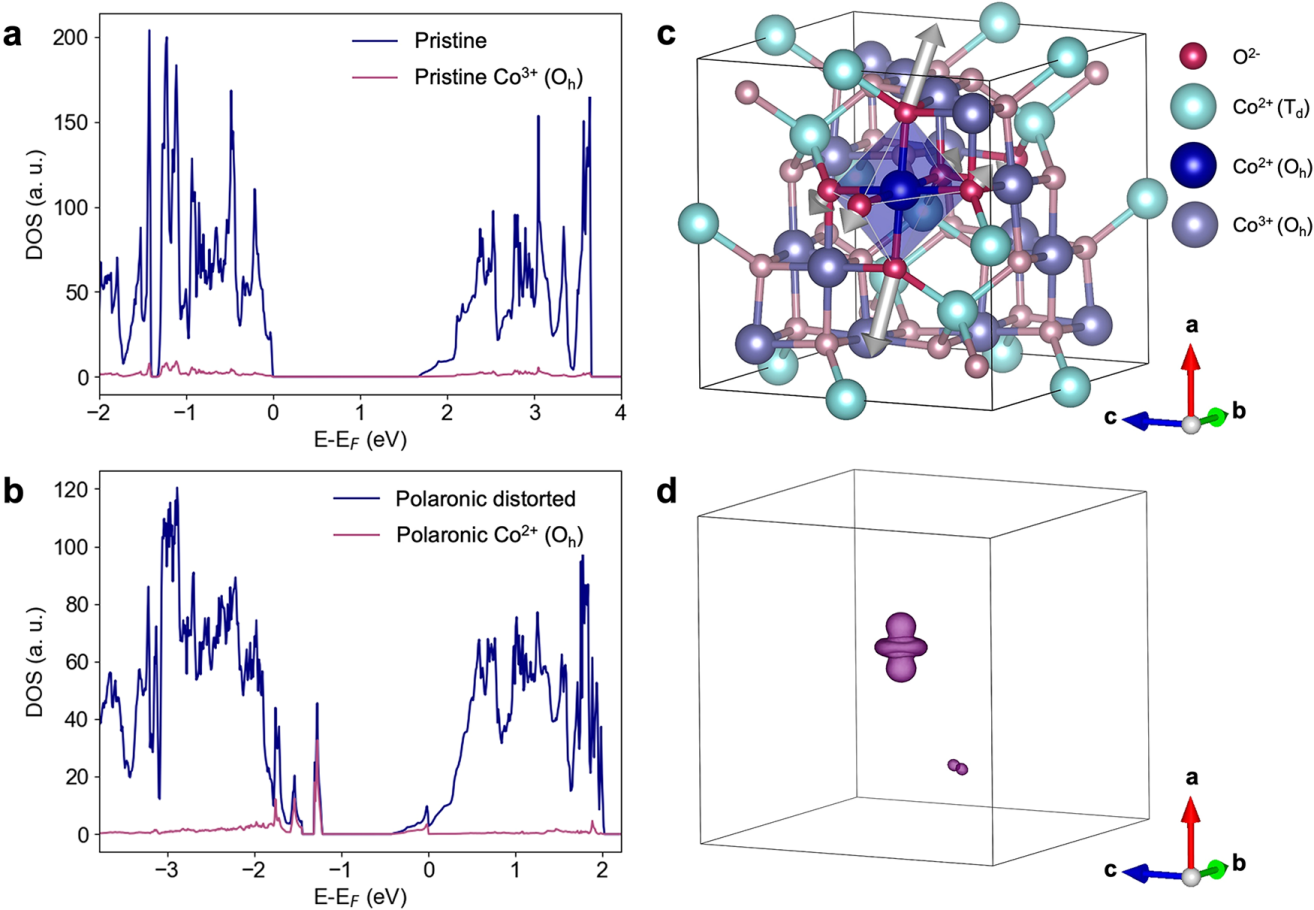
Constrained DFT calculation of Jahn–Teller polaron formation in spinel Co_3_O_4_. Electron DOS of the (a) pristine and (b) polaronic structures across the band gap. The purple trace highlights the 3d orbital contributions of the (a) pristine Co^3+^(*O_h_*) site and (b) polaronic Co^2+^(*O_h_*) center, leading to the appearance of occupied states within the band gap. (c) Structural relaxation of the Co^2+^(*O_h_*) polaronic center toward the supercell energy minimum, involving a dominant displacement of the oxygens along the *a*-axis. (d) Isosurface plot of the excess electron localized on the 3d_*z* ^2^_ orbital of the Co^2+^(*O_h_*) site (purple: spin up), obtained as the difference of the spin density of the polaronic supercell in the presence and absence of the electron polaron charge.

## Discussion

Our combined experimental and theoretical results attribute Jahn–Teller polaron formation in Co_3_O_4_ to the LMCT transition from O^2–^ to Co^3+^ sites. This induces a local symmetry breaking of the crystal driven by the lifting of the orbital degeneracy,^2^ modifying the electronic transition energies. In this framework, the 10.2 meV coherent oscillation, which is absent in the phonon dispersion of the structure at equilibrium, becomes Raman-active and modulates the dielectric function, thereby giving rise to a detectable coherent response in transient reflectivity.

Our constrained DFT results are consistent with prior computational work predicting the influence of polaronic states on the absorption spectrum of Co_3_O_4_.^23^ In that study, optical excitations involving localized polarons were distinguished from band-like transitions by applying a 1% uniaxial tensile strain along the [100] direction. This external perturbation lifted the 3-fold degeneracy of the polaronic states, preferentially stabilizing the JT-distorted configuration elongated along [100] by approximately 5 meV relative to distortions along the [010] or [001] directions.

We also note that activation of Jahn–Teller modes has been reported in Prussian blue analogue compounds following ultrafast excitation of Co^3+^(*O_h_*) centers, leading to the formation of distorted Co^2+^ sites.^42^ Moreover, a similar mechanism in spinel Co_3_O_4_ has been proposed in a recent ultrafast optical nanoscopy study, which observed a suppression of charge-carrier diffusion following LMCT excitation, an effect attributed to efficient photocarrier localization and the emergence of polaronic states.^43^

This makes JT polarons a compelling microscopic description of the Co_3_O_4_ photodynamics that is fully consistent with all our experimental observations and DFT simulations. Indeed, based on infrared spectroscopy measurements^44,45^ and DFT calculations,^46^ the 10.2 meV coherent mode that we detect can neither be an acoustic nor an infrared-active mode or an inactive optical phonon of the cubic spinel *Fd*3̅*m* structure (Section S3 of the SI). Furthermore, the oscillation cannot be ascribed to magnons or bimagnons, since it persists up to room temperature and it is not strongly overdamped,^38,47–52^ see Figure 4c and Figure S24 of the SI. By fitting the oscillations with a damped cosine function, we obtain an average phase φ = 0.15 ± 0.10 rad and a damping time *t*_D_ = 1.5 ± 0.3 ps (see Figure S22 of the SI), which is in contrast with a possible overdamping process.

Moreover, these oscillations cannot be ascribed to elementary excitations arising from a hidden phase of the system, e.g., charge order with a critical temperature between RT and 4.2 K.^53^ If this were the case, one would anticipate a softening of the oscillatory response approaching the critical temperature, or a quenching of the FT frequency/amplitude when the fluence exceeds a critical value.^54^ This is not detected in our data (Figure 4c), even for the highest photocarrier density value of 20.9·10^20^ cm^–3^ (Figure S25 of the SI).

The 10.2 meV mode can likewise be excluded as originating from a photoinduced structural phase transition^55^ (Section S6 of the SI). The possibility to reach the Co_3_O_4_ orthorhombic *Fddd*, monoclinic *C*2/*m* and monoclinic *P*2_1_/*c* phases was shown with the application of a static pressure in steady-state experiments.^56^ However, under such circumstances, oscillatory modulations should be observed, given by coherent optical phonons activated in the excited structural phase. Moreover, a transition to the purely *P*2_1_/*c* monoclinic structure would require an increase of the coordination number of the Co^2+^ sites from four to six, making this scenario unlikely.

The high purity of our sample, comparable to single crystals, leads to exclude the contribution of vacancies to the observed dynamics (Sections S1.4, S1.5, and S3 of the SI). Self-trapped excitons are also ruled out due to their higher energy instability with respect to polarons, as demonstrated computationally.^23^ Ultimately, we also exclude a photothermal process driving the phase transition, as the transient lattice temperature does not change within the time scale of the observed coherent dynamics (see Section S5.2 of the SI). Other in-depth observations and details behind the reasoning presented here are further elaborated in the SI.

## Conclusions

In this work, we reported state-selective excitation in spinel Co_3_O_4_, a prototypical correlated TMO characterized by a complex interplay of electronic, spin, and lattice degrees of freedom. Excitation at 3.10 eV targets *O*_*h*_ Co^3+^ ions and it unravels the microscopic mechanism of Jahn–Teller polaron formation. Specifically, the charge transfer process between O^2–^ and Co^3+^ octahedral sites leads to a local crystal symmetry breaking along the *a*-axis due to the JT effect, associated with charge localization on the 3d_*z* ^2^_ orbital at the Co­(*O_h_*) site, with the formation of stable polaronic in-gap localized states. The JT interaction, in turn, leads to a change of the electronic transitions’ energy in the system through deformation potential and magnetoelastic coupling. On the other hand, the state-selective 1.55 eV excitation reveals the specific modulation of the *T*_*d*_ sites of spinel Co_3_O_4_, due to the on-site d-d transition primarily affecting the Co^2+^ centers, via an ordinary electron–phonon coupling characterized by a symmetry-retaining lattice distortion. Our approach promises broad applicability to a host of systems in which JT polaron physics has been predicted but, to date, not yet experimentally verified. More broadly, it enables local engineering of TMOs electronic and structural properties with ultrafast light pulses, marking the route toward on-demand tailoring of functional responses in quantum materials.

## Supplementary Material



## Data Availability

The processed data shown in this manuscript are available in the Supporting Information. All data supporting the conclusions in the paper are present in the main text and in the SI. Raw data are available in the following repository: 10.5281/zenodo.14782648.

## Notes

Sample synthesis and characterization; experimental, analysis and computational methods; Co_3_O_4_ phonons; assignment of Co_3_O_4_ optical transitions; Co_3_O_4_ transient reflectivity; assignment of the 10.2 meV collective mode; Figures: S1 to S28; Tables: S1 to S9 (PDF)

## ◆◆

Centre for Free-electron Laser Science, Deutsches Elektronen-Synchrotron, Notkestraße 85, Hamburg 22607, Germany
