# Supplementary material for: Ultrafast Formation of Jahn–Teller Polarons Revealed by State-Selective Excitation in Correlated Spinel Co3O4
Source: J Am Chem Soc. 2026 Apr 1;148(18):18839–48. doi: 10.1021/jacs.5c23346 (PMC13185109; doi:10.1021/jacs.5c23346)
Supplement: Supplementary file 1 [file ja5c23346_si_001.pdf]

Supporting Information

Ultrafast Formation of Jahn-Teller Polarons Revealed by State-Selective Excitation in Correlated Spinel Co_3O_4

Simone Restelli[1,2]†, Oliviero Cannelli[3,4]†‡, Nicola Colonna[5], Carmelo Grova[1], Paolo Usai[3], Michele Puppin[3,4], Mounir Mensi[6], Francesco Barantani[7], Yue Meng[7], Jérémie Teyssier[8], Malte Oppermann[3,4,9], Francesco Pennacchio[3], Camila Bacellar[3,4,10], Jérémy R. Rouxel[3,4,10,11], Ludmila Diniz Leroy[3,4,10], Oleg Dogadov[3,4], Natacha Ohannessian[12], Daniele Pergolesi[12,13], Pietro Galinetto[2], Przemysław Piekarz[14], Andrzej Ptok[14], Swati Chaudhary[15], Gregory A. Fiete[15,16], Majed Chergui[3,4,17], Edoardo Baldini[7], Giulia F. Mancini[1]*

[1] Laboratory for Ultrafast X-ray and Electron Microscopy (LUXEM), Department of Physics, University of Pavia, Pavia 27100, Italy.
[2] Department of Physics, University of Pavia, Pavia 27100, Italy.
[3] Lausanne Centre for Ultrafast Science (LACUS), École Polytechnique Fédérale de Lausanne, Lausanne 1015, Switzerland.
[4] Laboratory of Ultrafast Spectroscopy (LSU), École Polytechnique Fédérale de Lausanne, Lausanne 1015, Switzerland.
[5] PSI Center for Scientific Computing, Theory and Data, Paul Scherrer Institute (PSI), Villigen 5232, Switzerland.
[6] Institute of Chemical Sciences and Engineering (ISIC), X-Ray Diffraction and Surface Analytics Platform (XRDSAP), École Polytechnique Fédérale de Lausanne, Sion 1950, Switzerland.
[7] Department of Physics, The University of Texas at Austin, Austin, Texas 78712, USA.
[8] Department of Quantum Matter Physics, University of Geneva, Geneva 1211, Switzerland.
[9] Department of Chemistry, University of Basel, Basel 4056, Switzerland.
[10] SwissFEL, Paul Scherrer Institute (PSI), Villigen 5232, Switzerland.
[11] Chemical Sciences and Engineering Division, Argonne National Laboratory, Lemont, Illinois 60439, United States.
[12] PSI Center for Neutrons and Muons Sciences, Paul Scherrer Institute (PSI), Villigen 5232 PSI, Switzerland.
[13] PSI Center for Energy and Environmental Sciences, Paul Scherrer Institute (PSI), Villigen 5232 PSI, Switzerland.
[14] Institute of Nuclear Physics, Polish Academy of Sciences, Kraków 31-342, Poland.
[15] Department of Physics, Northeastern University, Boston, Massachusetts 02115, USA.
[16] Department of Physics, Massachusetts Institute of Technology, Cambridge, Massachusetts 02139, USA.
[17] Elettra-Sincrotrone Trieste S.C.p.A., S.S. 14 km 163,5 in Area Science Park, Basovizza, Trieste 34012, Italy.

*Corresponding author: giuliafulvia.mancini@unipv.it
† These authors contributed equally to this work.
‡ Current affiliation: Centre for Free-electron Laser Science, Deutsches Elektronen-Synchrotron, Notkestraße 85, Hamburg 22607, Germany.

<u>**Table of Contents**</u>

1. Sample synthesis and characterization

- 1.1 Sample synthesis
- 1.2 X-ray absorption spectroscopy
- 1.3 Static X-ray diffraction
- 1.4 Electron diffraction
- 1.5 X-ray photoemission spectroscopy

2. Experimental, analysis and computational methods

- 2.1 Broadband transient reflectivity
- 2.2 Data analysis
- 2.3 Computational methods and DFT calculations

3. Co_3O_4 phonons

4. Assignment of Co_3O_4 optical transitions

5. Co_3O_4 transient reflectivity

- 5.1 Transient reflectivity at RT
- 5.2 Transient reflectivity as a function of pump fluence and temperature
- 5.3 Coherent response
  - 5.3.1 Phase and damping time
  - 5.3.2 1.55 eV photoexcitation as a function of temperature and fluence
  - 5.3.3 3.10 eV photoexcitation as a function of temperature and fluence

6. Assignment of the 10.2 meV collective mode

- 6.1 Acoustic phonons
- 6.2 CoO phonons
- 6.3 Sapphire substrate phonons
- 6.4 Single magnons and bi-magnon excitations
- 6.5 Pump-induced structural transition

Figures: S1 to S28
Tables: S1 to S9

1. Sample synthesis and characterization

1.1 Sample synthesis

The sample consists of a 27 nm Co_3O_4 thin film epitaxially grown by pulsed laser deposition[1] on a [0001]-oriented sapphire (Al_2O_3) single crystal substrate. The sapphire substrate was chosen due to the matching of the unit cell dimensions and space groups of Al_2O_3 and Co_3O_4, which favour an epitaxial growth along the [111] direction.

The substrates were cleaned in an ultrasonic bath using de-ionized water, acetone and isopropanol. A resistive heater was used to heat the substrate to the selected deposition temperature of 650 ^oC. The substrates were mounted into a vacuum chamber, with a base pressure of about 10^{-8} mbar, onto a stainless steel plate using Ag paste to provide the required thermal contact. A small spot of Platinum (Pt) paste was painted near the edge of the substrate. A pyrometer pointing at the Pt spot was used to read out the deposition temperature using the black-Pt emissivity value of 0.97. A high purity O_2 partial pressure of $1.3 \cdot 10^{-3}$ mbar was set prior to the deposition and kept constant during the growth. A Co_3O_4 pellet fabricated in our laboratory was used as the target for pulsed laser deposition. A 248 nm KrF excimer laser was focused onto the target surface over a spot of about 1.5 mm^2. The energy density at the target was set at about 2.8 $J \cdot cm^{-2}$. The target to substrate distance was set at 40 mm. The laser was operated at a repetition rate of 2 Hz. The deposition rate was calibrated by X-ray reflectometry. With the selected set of deposition parameters, we measured a deposition rate of 0.015 nm per laser pulse. After the growth, the samples were post-annealed *in situ* at the deposition temperature for 20 minutes at an O_2 partial pressure of 0.1 mbar.

The sample purity and crystallographic orientation was assessed through X-ray absorption spectroscopy, X-ray diffraction and electron diffraction.

1.2 X-ray absorption spectroscopy

Steady-state X-ray absorption spectroscopy (XAS) measurements on Co_3O_4 were performed at the MicroXAS beamline of the Swiss Light Source (SLS). The sample and detector were respectively at 45° and 90° with respect to the incoming X-rays and the XAS signal was collected in fluorescence geometry. The Co K absorption edge (7.700-7.780 keV) was probed using a Ketek detector with a resolution of about 150 eV and a Si (311) crystal monochromator. This resolution allows isolating the Co fluorescence lines while suppressing the elastic scattering signal. The intensity was corrected by the incoming X-ray flux and the energy scale was calibrated using a Co foil. In the thin-sample limit[2], which applies to our 27 nm Co_3O_4 thin film, the collected signal is proportional to the XAS absorption coefficient. Figure S1a compares the measured Co_3O_4 XAS spectrum with literature results from Bordage *et al.*[3]. The spectra were normalized to their maximum intensity and shifted vertically by an arbitrary offset for clarity.

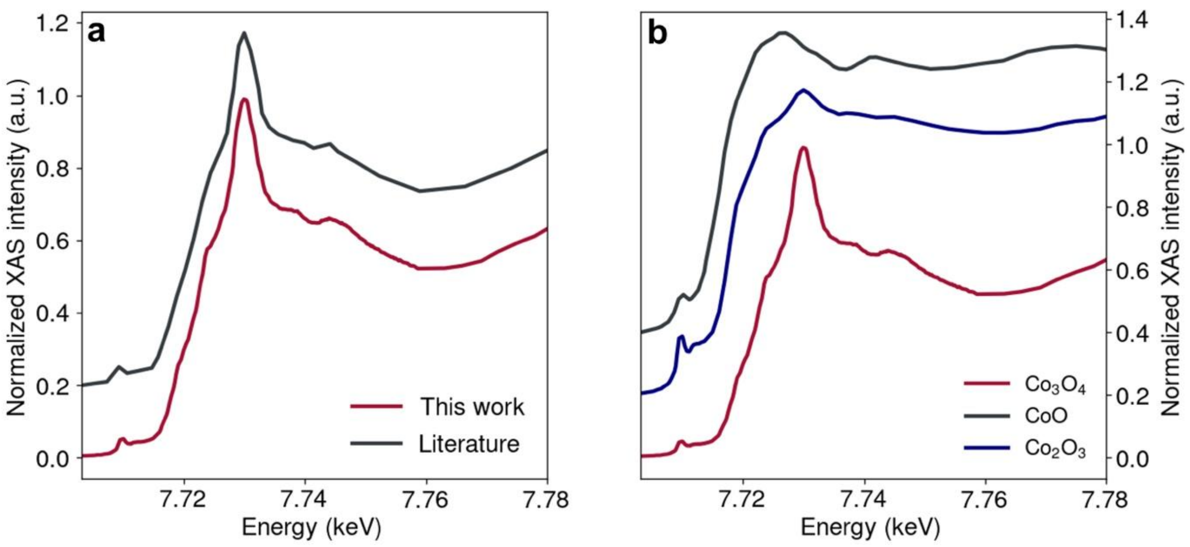

Figure S1. (**a**) XAS spectrum of spinel Co_3O_4 film (red line) compared with literature measurements[3] (black line). (**b**) XAS spectra of Co_3O_4 (red line) and reference samples including only Co^{2+} (CoO, black line) and Co^{3+} (Co_2O_3, blue line) centres. The spectra are normalized by their maximum of intensity and shifted by an arbitrary offset along the vertical axis.

A good agreement between the two spectra is observed in the pre-edge, main-edge, and post-edge regions, confirming the sample quality. The chemical purity of the spinel Co_3O_4 thin film was further verified, and the presence of cobalt oxides with other stoichiometries was excluded by measuring the reference spectra of two compounds (Figure S1b): CoO (black line) and Co_2O_3 (blue line). The differences between Co_3O_4 and the reference compounds are most evident in the rising-edge energy position, which is blue-shifted for Co_3O_4.

1.3 Static X-ray diffraction

The spinel Co_3O_4 used in this study was epitaxially grown along the [111] out-of-plane direction on a [0001]-oriented sapphire (Al_2O_3) single crystal. The X-ray diffraction (XRD) analysis shown in Figure S2a indicates a broadening of the diffraction peak due to size-effect interference fringes. Figure S2b is a representative X-ray reflectometry measurement used to calibrate the deposition rate, indicating that the film possesses a well-defined, flat surface and interface. Grazing incidence XRD of Co_3O_4 was performed at the Surface Diffraction end station of the Material Science beamline of SLS. The setup is shown in Figure S3a. The sample was mounted on a hexapod stage and oriented vertically, with an incidence angle of 0.33° relative to the X-ray beam. An X-ray photon energy above the Co absorption edge (9.3 keV) was selected to avoid collecting fluorescence background arising from resonant excitation of the Co centers (see Figure S1). The diffracted signal was detected with a Pilatus 100k detector. We mapped the position of the Bragg reflections composing the Ewald sphere in the three-dimensional space, identifying separately

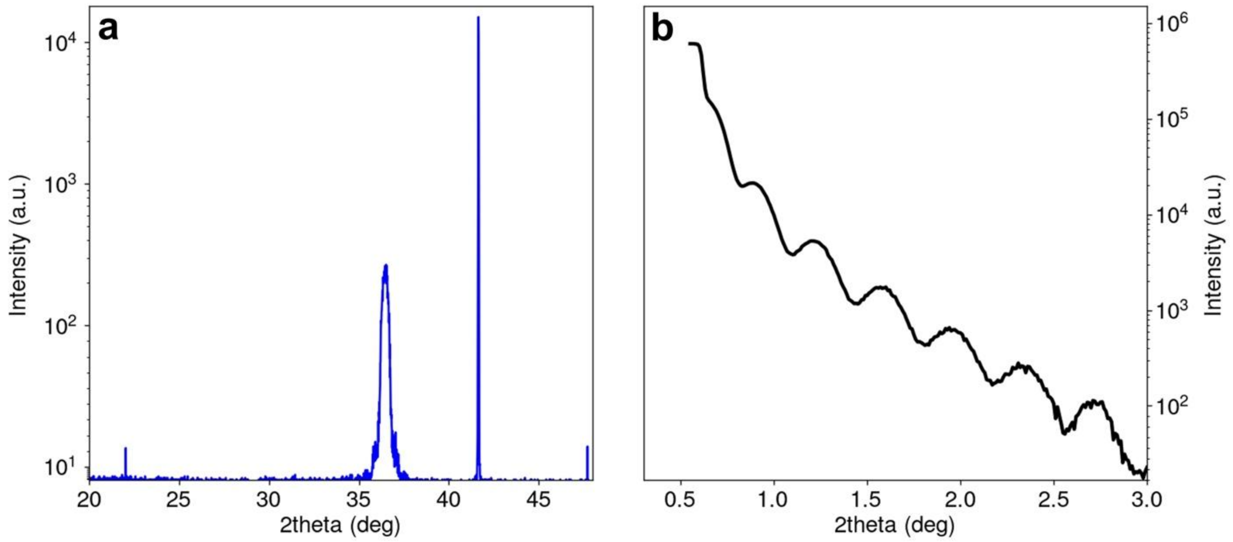

Figure S2. (**a**) XRD of the 27 nm spinel Co_3O_4 film grown on a sapphire crystal. (**b**) X-ray reflectivity of the sample, characterized by a well-defined flat surface and interface.

the diffraction peaks of the film and of the substrate, by determining the orientation matrix of the sample. For the Co_3O_4 lattice parameters, we used a = b = c = 8.15185 Å and α = β = γ = 90°, consistently with the $Fd\bar{3}m$ structure of the system. Figure S3b shows a typical diffraction peak, (311), characterized by a circular shape covering about 28×30 pixels. The diffraction intensity was maximized by rotating the sample about the in-plane angle ("rocking curve" in Figure S3c).

1.4 Electron diffraction

The surface crystallinity and the homogeneity of the sample were characterized with an experimental set-up for Reflection High-Energy Electron Diffraction (RHEED)[4]. We used an electron beam of 30 keV and collected the signal with a CCD camera having an active area of 25 mm (1340×1300 pixels, pixel size=21×21 μm^2) positioned 160 mm from the sample. The brightest peak was observed 5.544 mm above the position of the direct beam. Using the out-of-plane $[111]$ and the in-plane $[\bar{1}10]$ vectors, determined from grazing-incidence XRD measurements, the peaks in the pattern of Figure S4 were assigned by comparison with simulations, performed using CrystalMaker Software Ltd[5]. The experimental data show 11 intense peaks with a streaked shape. Satellite peaks and modulated or inclined streaks have not been observed, suggesting that our Co_3O_4 thin film is characterized by a high level of epitaxial crystallinity and a flat surface with domains having a small degree of rotation.

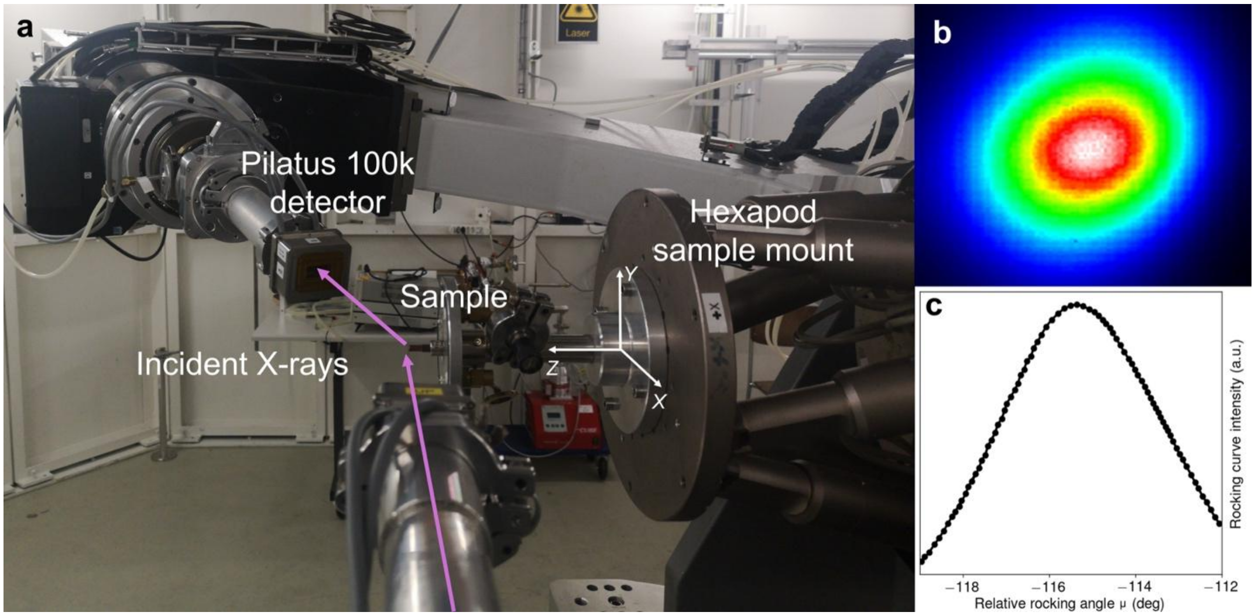

Figure S3. (**a**) XRD experimental layout of the Material Science beamline at SLS. The sample was vertically oriented in grazing incidence geometry with respect to the incoming X-ray beam. A Pilatus 100k detector is set in the geometry corresponding to the (311) diffraction peak. (**b**) (311) diffraction peak. (**c**) Rocking curve of the (311) peak.

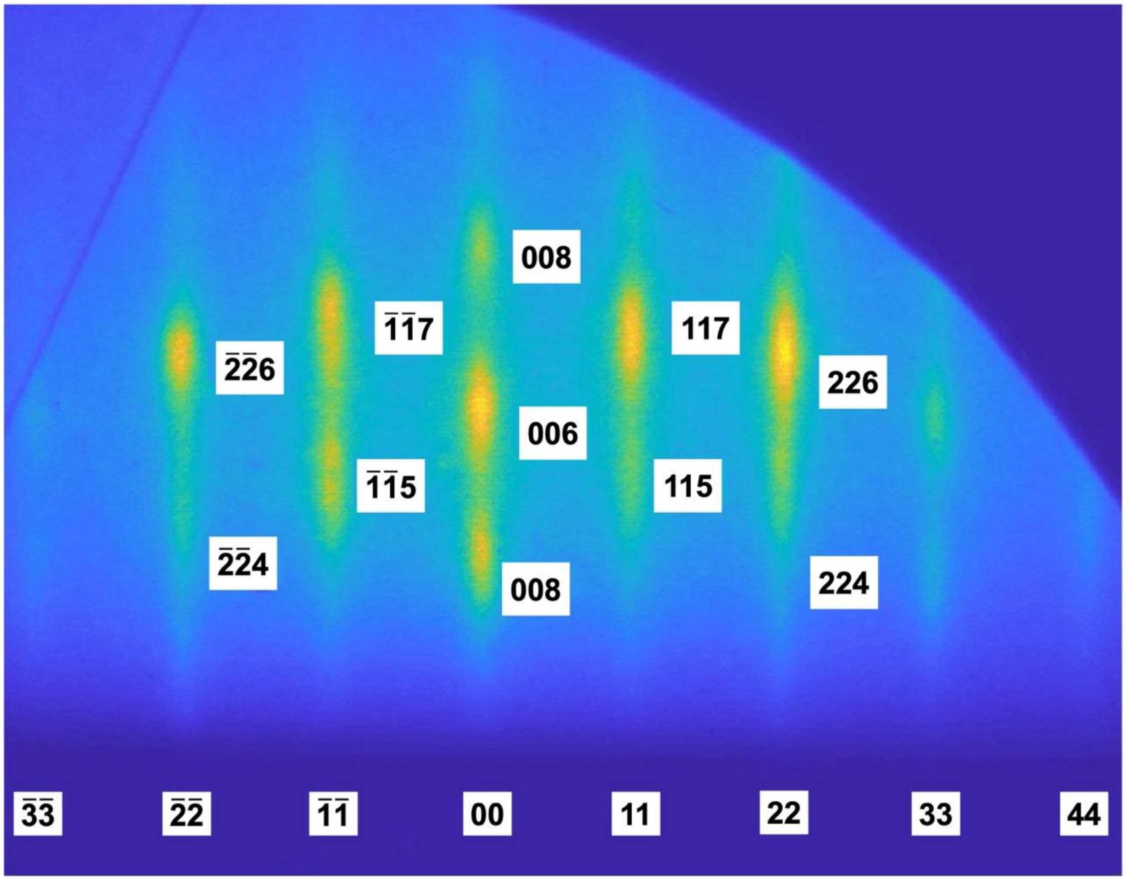

Figure S4. Reflection High-Energy Electron Diffraction pattern of spinel Co_3O_4.

1.5 X-ray photoemission spectroscopy

The surface quality of the spinel Co_3O_4 thin film was verified by performing X-ray photoemission spectroscopy (XPS) using an Axis Supra instrument. The experimental results of the Co 2p spectrum from our thin film show an excellent agreement with literature results for bulk spinel Co_3O_4[6], indicating negligible surface reconstruction effects.

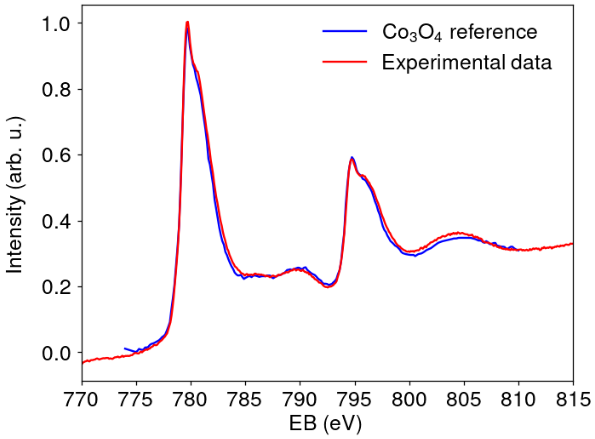

Figure S5. XPS measurements of spinel Co_3O_4 reported in the literature (blue curve)[6] and our thin film (red) after normalization by the maximum of each curve, linear background subtraction, and 5 eV shift.

2. Experimental, analysis and computational methods

2.1 Broadband transient reflectivity

Transient reflectivity measurements were performed using a Ti:sapphire oscillator (Halcyon, KMLabs), pumped by a continuous wave Nd:YVO_4 laser (VERDI, Coherent), delivering sub-50 fs pulses centered at 1.55 eV at a repetition rate of 80 MHz to seed a cryogenically cooled regenerative amplifier (Wyvern500, KMLabs). The latter is pumped by three Q-switched Nd:YVO_4 pump lasers (Photonics Industries) and it operates at 20 kHz. The output of the amplifier consists of 60 fs pulses of 0.6 mJ centered at 1.55 eV with a shot-to-shot energy fluctuation below 0.1% rms. A 10 μJ portion of the p-polarized output is split into pump and probe branches with an 80:20 ratio by an ultrafast beam splitter. The pump pulse is temporally delayed with respect to the probe by a motorized delay line and focused with a 20 cm focal-length spherical mirror into a 0.5 mm-thick type I β-BaB_2O_4 (BBO) crystal to generate 3.10 eV pulses. A 100-blades mechanical chopper is placed close to the focus position and it operates at 10 kHz, phase-locked to the laser repetition rate (20 kHz). The beam is collimated with another 20 cm spherical mirror and, when the pump is frequency doubled, the residual 1.55 eV intensity is removed with 4 harmonic separators. The pump is then focused into the sample at 45° incidence angle with respect to the surface normal direction. In the probe branch, supercontinuum white light is generated by focusing the laser fundamental frequency into a 2 mm-thick CaF_2 crystal, which is continuously moved at 10 Hz along the vertical direction and scanned along the orthogonal direction of the

beam propagation with a motorized stage to prevent short and long term photoinduced degradation of the crystal. A pair of 90° off-axis parabolic mirrors is used to collimate and focus the probe on the sample with an angle of incidence of 22°. The residual intensity of the 1.55 eV in the probe arm is removed before the sample with a glass filter. The dimension of the pump and probe beams at the sample position are 150×150 μm^2 and 90×80 μm^2 in full width at half maximum (FWHM), respectively. The reflected probe output is collected with a 2 m long multi-mode optical fibre into a spectrograph where the broadband pulse is dispersed by a 150 gr/mm holographic grating. The resulting spectrally-resolved pumped and unpumped signal intensities are measured in shot-to-shot detection mode with a complementary metal-oxide semiconductor (CMOS) array detector (Hamamatsu S11105, 512 pixels, 12.5×250 μm^2 pixel size) synchronized to the data acquisition system. A gated signal is delivered by a triggering card that synchronizes the laser amplifier signal (20 kHz) with the 11 MHz master clock of the 16-bit analog-to-digital converter (ADC) and triggers the CMOS readout operation. A replica of this gated trigger is halved and used to phase-lock the chopper at 10 kHz to the laser amplifier output. For each time delay, 20 000 consecutive shots are recorded, sorted into *pumped* (p) and *unpumped* (u) events, and averaged. The *ΔR/R* signal is computed as:

$$\frac{\Delta R(\omega,t)}{R} = \frac{R_p(\omega,t) - R_u(\omega,t)}{R_u(\omega,t)} \tag{S1}$$

where ω and t correspond to the spectrally resolved frequency of the probe and the time delay between the pump and the probe, respectively. The transient reflectivity signal is collected as a function of the time delay and probe energy. The signal-to-noise ratio (SNR) of the resulting 2D maps is increased by averaging ∼20 consecutive scans. The frequency calibration is performed by measuring the absorption spectrum of a holmium solution and the corresponding pure solvent (Hellma). Since the supercontinuum probe is not dispersion-compensated, the transient reflectivity maps are chirp-corrected in post-processing. To define the parameters for the group velocity dispersion (GVD) correction, transient reflectivity measurements on the sapphire substrate were performed. An optical cryostat (Optistat, Oxford instruments) with a lower temperature limit of 1.5 K was installed in the setup for the transient reflectivity measurements as a function of temperature. The cryostat is equipped with a copper cold finger sample holder, a heating resistance and a thermocouple connected to an external temperature controller (Oxford instruments). The vacuum system consists in a turbomolecular pump (Pfeiffer) and a backup scroll pump (Busch). The helium flux is regulated through a membrane pump (Pfeiffer) and a gas flowmeter (Oxford instruments).

2.2 Data analysis

The data analysis was carried out through a combination of Python scripts and the open-source OPTIMUS software[7]. The calibrated transient reflectivity data were imported in OPTIMUS, and

the substrate measurements were used to define the white light chirp through a second order polynomial fitting function of the coherent artifact signal. For each dataset, we performed a global fit as a function of time delay with a sum of exponentials convoluted with a gaussian instrument response function (IRF)[7]:

$$S_\omega(t) = \sum_{i=1}^{n} A_i(\tau_i, \omega) \cdot e^{-\frac{t}{\tau_i}} * IRF(t) \tag{S2}$$

where $A_i(\tau_i,\omega)$ is the frequency-dependent amplitude of the exponential decay characterized by the τ_i time constant and $* IRF(t)$ indicates the convolution of each component with a gaussian function. This method allows the simultaneous fitting of all probe energies employed in the experiment and it returns the global time constants of the system.

The coherent response was isolated by subtracting the global multiexponential fitting function from the transient reflectivity data to remove the incoherent response. The resulting traces were spectrally averaged in the probe energy ranges 1.70-1.80 eV. A polynomial function was subtracted to remove the contributions of overdamped acoustic phonons near time zero. The coherent response was analysed by applying a fast Fourier transform (FT), with zero-padding and Kaiser-Bessel windowing[8]. The FT and oscillation traces were respectively fitted with Lorentz and sinusoidal functions.

2.3 Computational methods and DFT calculations

We performed density functional theory (DFT) calculations for spinel Co_3O_4 by optimizing the crystal and electronic structures using the projector augmented-wave method within the generalized gradient approximation (GGA) implemented in the VASP program[9]. The local electron interactions in the Co(*3d*) states were included using the DFT+U method[10] with the effective Coulomb parameter U_{eff} = 3.3 eV in order to reproduce the experimental band gap value of 1.6 eV[11], getting a lattice constant of a=8.136 Å (Figure S6). The cell parameters were taken from the literature[12] and their full relaxation was performed in the crystallographic cell of the $Fd\bar{3}m$ space group containing 56 atoms. Total energy convergence was achieved using the **k**-point 6×6×6 Monkhorst-Pack mesh and 520 eV energy cut-off for the plane waves (Figure S7). After optimization, the antiferromagnetic order was obtained with the magnetic moments on the Co^{2+} (T_d) sites (m = 2.75 m_B). The resulting electronic structure and partial electronic density of states (DOS) are reported in Figures S8 and S9. The calculations were performed also for the orthorhombic $Fddd$ and monoclinic $P2_1/c$ structures. The phonon dispersion curves were obtained using the direct method[13] implemented in the PHONON software[14] and are presented in this Supporting Information in Figures S15, S27, and S28 for the cubic, orthorhombic, and monoclinic symmetries, respectively. For the cubic structure, very similar results were obtained using the Phonopy code[15].

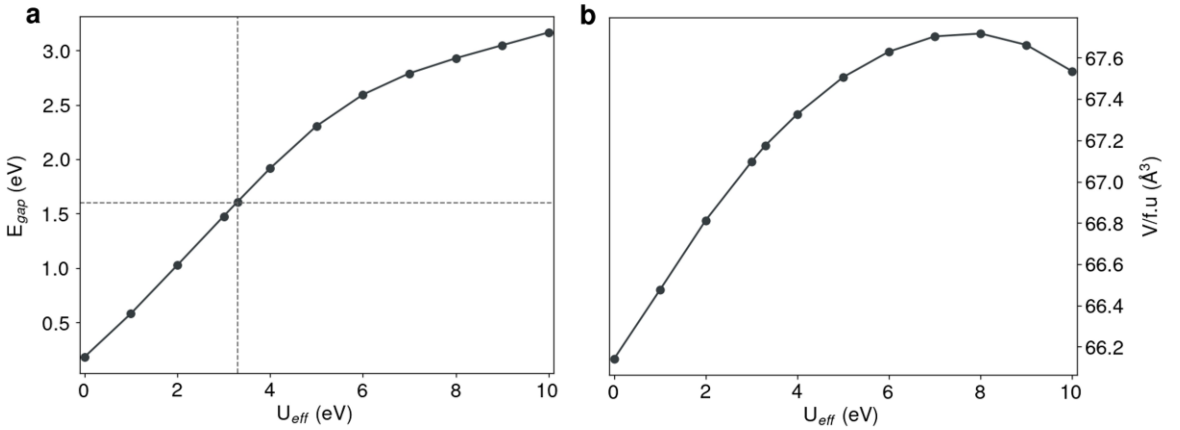

Figure S6. U_{eff}-dependence of (**a**) insulating gap and (**b**) system volume of spinel Co_3O_4 within DFT+U calculation. The experimentally observed gap (～1.6 eV)[11] is obtained for U_{eff}=3.3 eV.

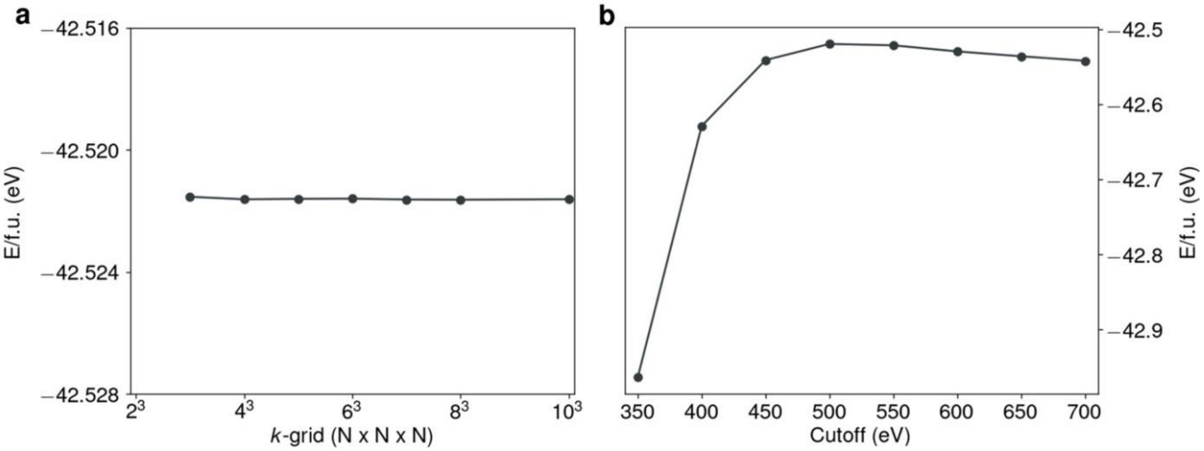

Figure S7. Energy convergence of spinel Co_3O_4 with respect to (**a**) **k**-grid (for cutoff 550 eV) and (**b**) cutoff energy (for **k**-grid 6×6×6).

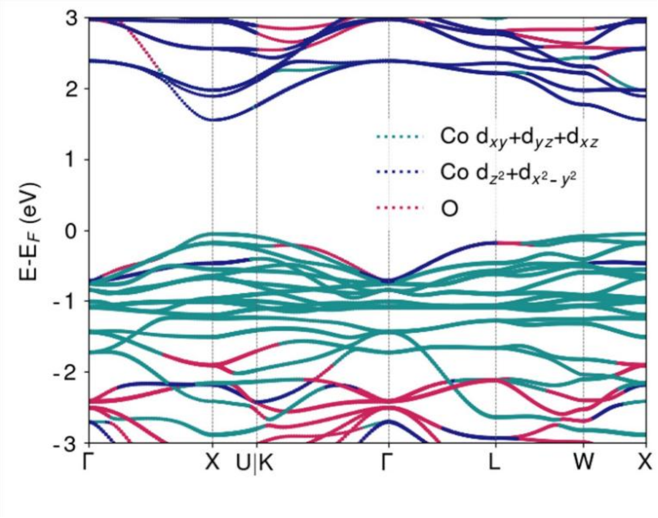

Figure S8. The electronic band structure of Co_3O_4.

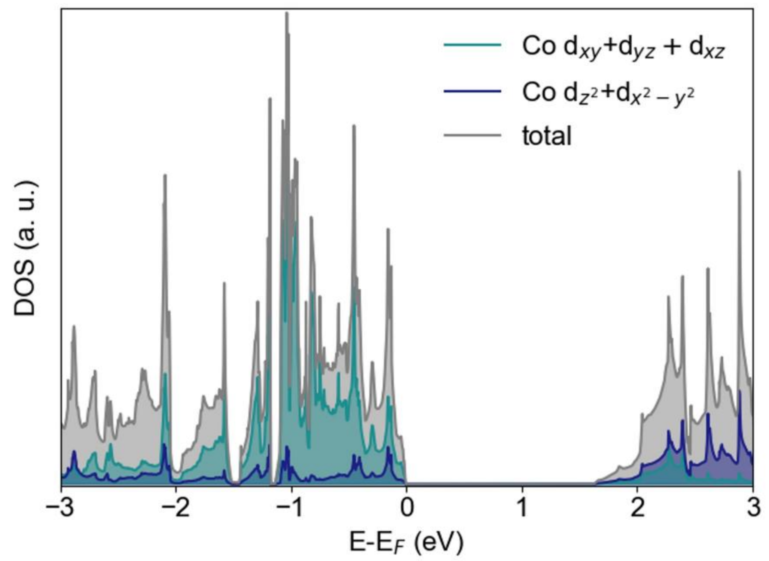

Figure S9. Total and partial electronic DOS of Co_3O_4.

Polaron calculations were performed using a constrained DFT+U approach in Quantum Espresso on a 2x2x2 supercell of spinel Co_3O_4. The simulation was initialized by using U_{eff}=3.8 eV, which provides the same band gap value of 1.6 eV used in the VASP program discussed above, and localizing a -1 charge on a specific Co(O_h) site, while constraining the total spin to +1.

Ultrasoft and PAW pseudopotentials consistent with the PBEsol exchange-correlation functional were used, with a plane-wave kinetic-energy cutoff of 50 Ry and a charge-density cutoff of 400 Ry. The BZ was sampled using a 4×4×4 Monkhorst-Pack **k**-point grid. Electronic and ionic relaxations were converged to thresholds of 2.8×10^{-9} Ry for the self-consistent field and 1.4×10^{-5} Ry and 1.0×10^{-5} Ry/bohr for total energy and forces, respectively.

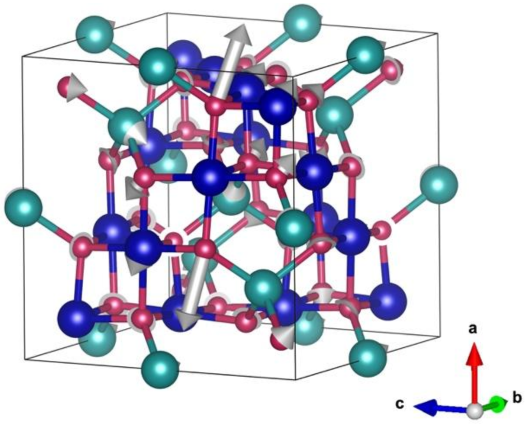

Figure S10. Vectors showing the structural changes of the pristine supercell into the relaxed polaronic structure.

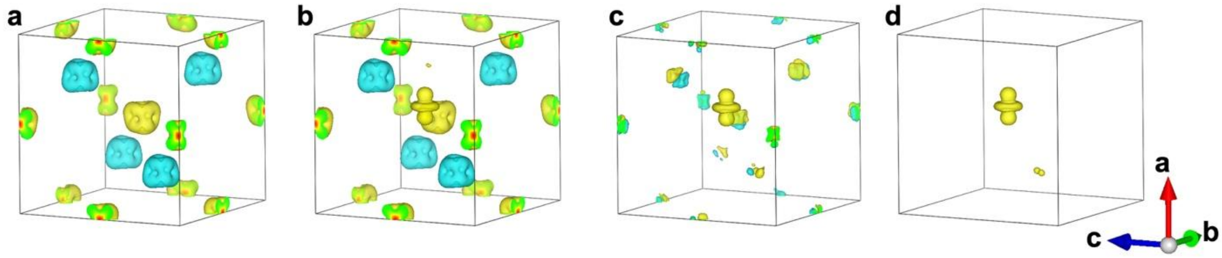

Figure S11. Isosurface plot of the spin density in Co_3O_4. (**a**) In the pristine system the spin is localised on the $Co^{2+}(T_d)$ sites with antiferromagnetic arrangement (yellow: spin up; light blue: spin down). (**b**) The polaronic structure shows an additional contribution due to the excess charge localised on the $3d_{z^2}$ of Co #20. (**c**) Spin density difference between the polaronic supercell in the presence of the electron polaron charge and of the pristine supercell. The residual contribution on the $Co^{2+}(T_d)$ sites is due to the different atomic position in the two supercells. (**d**) The latter is removed when subtracting the spin density of the same polaronic supercell in the presence and absence of the electron polaron charge.

The atomic changes occurring during the structural relaxation of the supercell towards the polaronic energy minimum are reported in Figure S10. Notably, these lattice changes are asymmetric, with a stronger distortion along the *a*-axis compared to the *b* and *c* directions, resulting in a local breaking of the cubic symmetry. Upon full structural relaxation the excess charge remains on the initial $Co(O_h)$, as shown in Figure S11 through the comparison of the spin densities for the pristine and polaronic systems. The phonon structure was computed for both pristine and polaronic supercells using the Phonopy code[15] and the resulting phonon DOS profiles are reported in Figure S12.

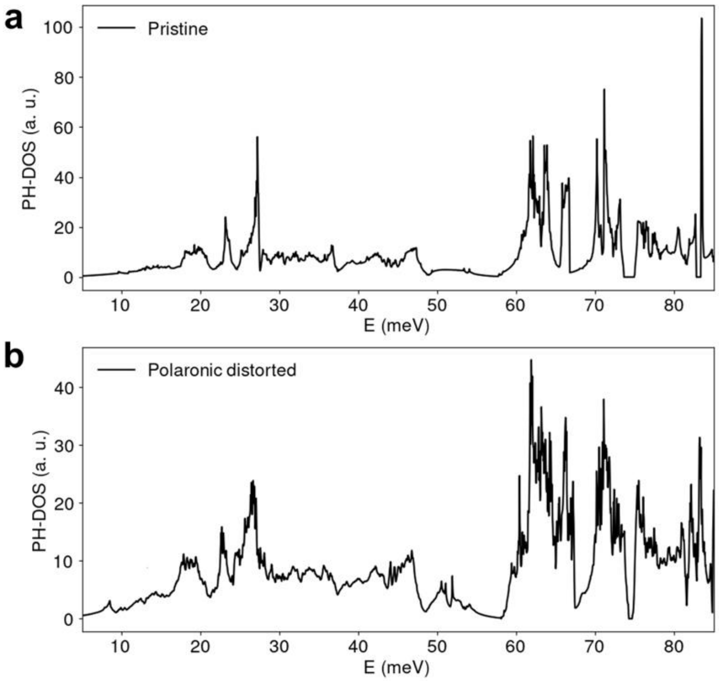

Figure S12. Phonon DOS of the (**a**) pristine and (**b**) polaronic Co_3O_4 structure.

Overall, our calculations support the formation of a Jahn-Teller polaron upon 3.10 eV photoexcitation. The ultrafast localization of the negative charge following ligand-to-metal charge transfer triggers a lattice distortion that locally breaks the symmetry of the system, causing a phonon softening that is probed in the time domain via transient reflectivity measurements at the Γ point.

3. Co_3O_4 phonons

The Co_3O_4 Bravais cell contains 14 atoms, giving rise to 42 phonon modes (3 acoustic and 39 optical)[16]. The irreducible representations of the O^7_h factor group are: $\Gamma = A_{1g} + E_g + 3T_{2g} + 4T_{1u} + 2A_{2u} + 2E_u + 2T_{2u} + T_{1u} + T_{1g}$, in agreement with a previous report[16]. Among these phonons, four are infrared-active with T_{1u} symmetry[17], five are Raman-active with $3T_{2g}$, E_g and A_{1g} symmetries[18], and the remaining modes are inactive. Figure S13 shows the far-infrared reflectivity spectrum of Co_3O_4 on a sapphire substrate (black dots), normalized to a gold film standard, together with its Lorentzian fit (red line).

The spectrum is dominated by the strong sapphire response due to the long penetration depth of the far-infrared photons. The contrast of the Co_3O_4 features is enhanced by dividing the reflectivity of the sample by the spectrum of pure sapphire, as reported in blue in Figure S13. The gray dashed lines mark the position of all infrared-active modes predicted by group theory at the energies: 26.6 meV, 48.9 meV, 70.5 meV, and 81.7 meV. The additional features at 61 meV and 75 meV are attributed to spurious normalization effects due to the differences between the thin film's substrate and the reference sapphire spectra. The experimental spontaneous Raman characterization of the system is reported in Figure 2b within the main text, showing all Raman-active phonons at the following energies (symmetries): 24.3 meV (T_{2g}), 59.9 meV (E_g), 64.8 meV (T_{2g}), 77.1 meV (T_{2g}), and 85.9 meV (A_{1g}). Also in this case, our results agree with both group theory predictions[18].

In Figure S14 we report the comparison of our experimental spectrum (red) with literature results for a Co_3O_4 single crystal (black)[18] and from the mineral database RRUFF (blue)[19]. Our sample shows the smallest FWHM for all peaks, as reported in Table S1. This is a confirmation of the thin film purity, comparable to Co_3O_4 single crystals and narrower than the sample reported in the RRUFF database, which contains natural contamination of other species such as copper, silicon and manganese, and the presence of oxygen vacancies.

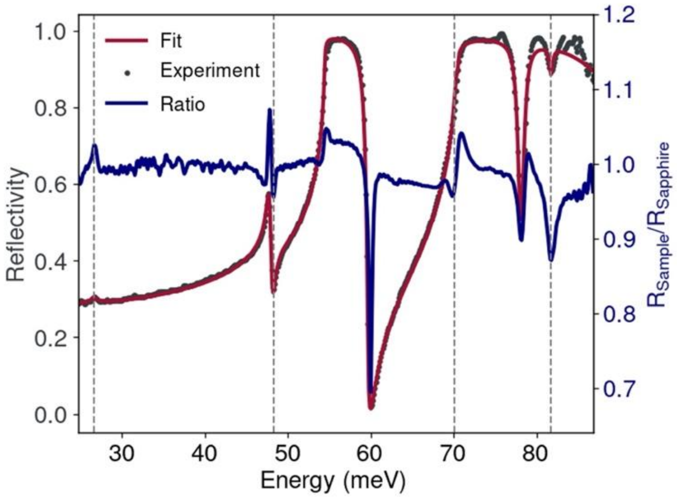

Figure S13. Reflectivity of Co_3O_4 on sapphire substrate in the far infrared (black dots) and the Lorentz fit (red line). The blue line corresponds to the ratio between the reflectivity of Co_3O_4 on sapphire and the pure sapphire substrate. The energy of the Co_3O_4 IR-active phonons is indicated with the black dashed lines.

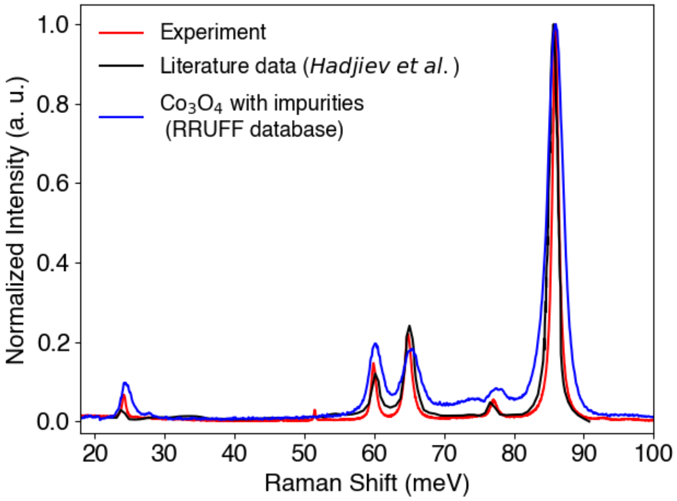

Figure S14. Raman spectrum of our Co_3O_4 sample (red) upon 2.33 eV excitation compared with literature single crystal one (black)[18] and RRUFF database data (blue)[19]. All spectra were normalized by their maximum intensity.

Peak energy, meV (symmetry)	FWHM, meV [our sample]	Literature (single crystal), meV [*Hadjiev, et al. Journal of Physics C: Solid State Physics 21.7 (1988): L199. DOI: 10.1088/0022-3719/21/7/007*]	RRUFF database sample, meV [*Lei, Zhilan, et al. Mineralogical Magazine 86.2 (2022): 346-353. DOI :10.1180/mgm.2022.27*]
24.3 (T_{2g})	1.0	1.2	1.9
59.9 (E_g)	0.8	1.5	2.0
64.8 (T_{2g})	1.2	1.5	2.9
77.1 (T_{2g})	1.0	1.0	2.2
85.9 (A_{1g})	1.0	1.4	2.4

Table S1. Comparison of FWHM of the Raman spectral features.

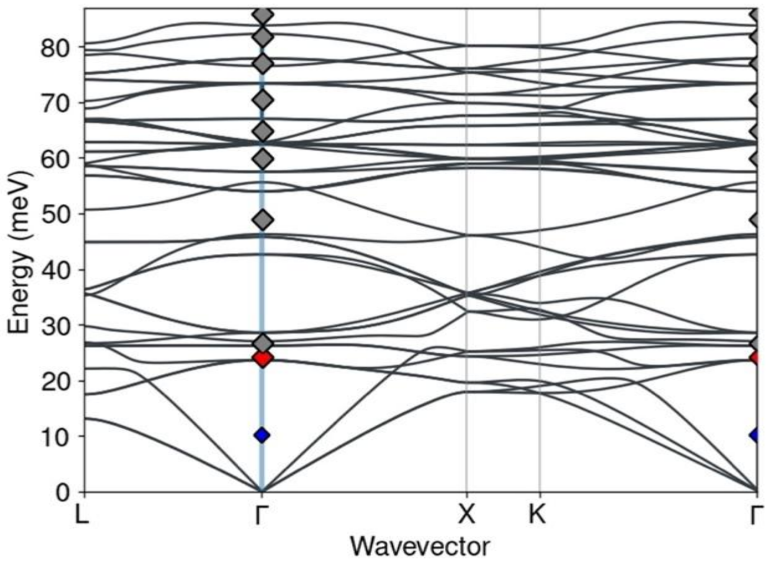

Figure S15. Phonon dispersion of the Co_3O_4 $Fd\bar{3}m$ lattice obtained through DFT calculations. The gray diamonds correspond to the phonon energies measured with steady-state experiments. The red and blue diamonds mark the energies measured in the transient reflectivity experiments upon 1.55 eV and 3.10 eV excitation.

DFT calculations for spinel Co_3O_4 are reported in Figure S15. The cell parameters were taken from the literature[12], and the full structural relaxation was performed in the crystallographic cell of the $Fd\bar{3}m$ space group containing 56 atoms. The phonon dispersion curves were obtained using the direct method implemented in the PHONON software[14]. The infrared, Raman, and inactive phonon modes at the Γ point are summarized in Table S2. The infrared- and Raman-active modes are in agreement with both our experiments and literature[17,18,20]. The 24.3 meV mode is a Raman active T_{2g} phonon, corroborating our interpretation of the Co_3O_4 coherent transient reflectivity response upon 1.55 eV pump excitation (see main text). The lowest inactive mode at the Γ point has an energy of 28.7 meV (T_{2u} symmetry). We note that no optical phonon was obtained at an energy scale of $\sim$10 meV.

**Raman-active modes**			
Experimental spectrum, Energy (meV)	DFT GGA+U, Energy (meV)	Literature[18], Energy (meV)	Symmetry
24.3	23.7	24.1	T_{2g}
59.9	57.5	59.8	E_g
64.8	62.6	64.7	T_{2g}
77.1	73.4	76.7	T_{2g}
85.9	82.3	85.7	A_{1g}

Infrared-active modes			
Experimental spectrum, Energy (meV)	DFT GGA+U, Energy (meV)	Literature[17,21], Energy (meV)	Symmetry
26.6	26.2	26.8 (26.8)	T_{1u}
48.9	45.8	48.6 (47.5)	T_{1u}
70.5	67.0	70.0 (71.0)	T_{1u}
81.7	77.9	81.5 (84.7)	T_{1u}

Inactive modes	
DFT GGA+U, Energy (meV)	Symmetry
28.6	T_{2u}
42.6	E_u
54.0	T_{1g}
55.6	A_{2u}
62.4	T_{2u}
62.9	E_u
76.5	A_{2u}

Table S2. Raman, infrared and inactive phonon modes in $Fd\bar{3}m$ spinel Co_3O_4: comparison between the energies obtained in our experiments, DFT simulations and literature.

The acoustic branch allows the extraction of the sound velocities along the principal crystallographic directions of the sample. The retrieved value is ~6 km/s, in agreement with literature studies on bulk and Young's moduli of Co_3O_4[22].

4. Assignment of optical transitions

The real and imaginary parts of the optical conductivity, σ_1 and σ_2, of spinel Co_3O_4 were obtained from ellipsometry measurements as a function of temperature using the RefFit software[23]. The following procedure was employed: (i) fitting the frequency-dependent refractive index of the sapphire substrate with a Lorentz model; (ii) simultaneously fitting both Co_3O_4 ellipsometric angles (Ψ and Δ) with a Lorentz model; (iii) applying the variational dielectric function (VDF) method to remove discrepancies between the Lorentz model and the sample response under the Kramers-Kronig constraint; and (iv) extracting optical quantities through built-in conversion functions. The RefFit software and the VDF method are described in detail elsewhere[23].

We fitted σ_1 spectra as a function of temperature with nine Gaussian functions. The results at room temperature (RT) and 7 K are reported in Figure S16a,b. Consistently with recent studies on Co_3O_4[24] and $ZnFe_2O_4$[25] thin films, we used gaussian functions for fitting σ_1 due to the

unphysical results obtained employing a Lorentz model. The Gaussian profile of the transitions is likely due to the homogeneous broadening caused by phonon dressing. The electronic transitions extracted from the Gaussian deconvolution were assigned in agreement with literature results for optical and resonant inelastic X-ray scattering experiments[26] and DFT calculations[24]. Figure S16c,d shows the σ_2 spectra at RT and 7 K, and the Kramers-Kronig curves of the fit obtained for σ_1.

At RT, we observe features at 0.82 eV, 0.92 eV, 1.27 eV, 1.63 eV, 1.76 eV, 2.71 eV, 3.63 eV, 4.68 eV, 5.74 eV (Figure S16a), in agreement with previous results[24,27]. Following the approach used in the literature[24], we ascribe the feature at 0.82 eV (feature 1) to on-site d-d transitions in the Co^{2+} cations with a final $^4T_1$ configuration[26]. We assign the 0.92 eV and 1.27 eV transitions (features 2 and 3) to intersite d-d excitations respectively involving the *e* and t_2 orbitals of Co^{2+} and the e_g orbitals of Co^{3+} sites. Another possible assignment for feature 3 is an on-site d-d transition of the Co^{3+} to the $^3T_{2g}$ state[26]. We attribute the feature 4 at 1.63 eV to a Mott-Hubbard (MH) intersite electron transfer between Co^{3+} t_{2g} and Co^{2+} t_2 sites, producing a Co^{4+} ion in the octahedral site and a Co^{1+} ion in the tetrahedral site[24,27].

The oscillator at 1.76 eV (feature 5) corresponds to a mixture of two on-site d-d transitions: (i) electron transfer from t_{2g} to e_g orbitals of Co^{3+} ($^1T_{1g}$) and (ii) an excitation of an *e* electron in Co^{2+}, reaching the $^2E_1$ electronic state. The 2.00-4.20 eV transitions (features 6 and 7) correspond to two ligand-to-metal charge transfer (CT) processes, respectively from O^{2-} *2p* to Co^{2+} t_2 orbitals and O^{2-} *2p* to Co^{3+} e_g orbitals[24,26]. We assigned the high-energy excitations at 4.68 eV (features 8) and 5.74 eV (feature 9, not shown) following literature results[24]. We note that the features labelled here as "4-7" in the main text are referred as "A-D". By lowering the temperature, a rearrangement of spectral weights is observed (Figure S16b). In particular, the lowest-energy absorption features are blue-shifted to 0.85 eV and 0.94 eV. The weaker broadening compared to RT makes the two features at 1.65 eV and 1.76 eV better resolved. No significant shifts are observed for the CT bands, which, however, become narrower. Table S3 summarizes our data at RT and 7 K and literature results[24,26].

Figure S17a,c report the central energy of the oscillators 4 and 6 as a function of temperature in the 7-120 K range. The peak energies exhibit shifts of ~8 meV between 7 K and 40 K, in contrast with the almost constant behaviour of the 40-120 K temperature range. The shifts below the Néel temperature (T_N) are ascribed to magnetoelastic coupling[28]. Oscillator 5 (Figure S17b), in contrast, exhibits a minor temperature shift of 1 meV, much smaller than the other two transitions.

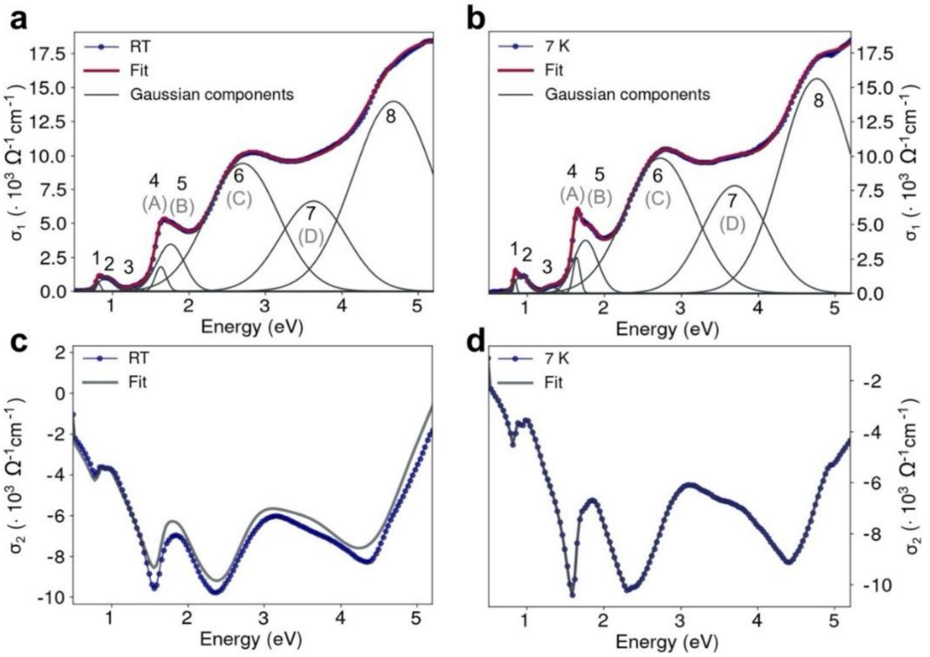

Figure S16. Real (absorptive) part of the optical conductivity σ_1 of Co_3O_4 (blue) plotted as a function of the photon energy at RT (**a**) and 7 K (**b**) across the antiferromagnetic transition (T_N ~ 30-40 K). The spectra are reported along with their fit (red) and gaussian deconvolution (black). The oscillators are labeled according to Table S2. Imaginary part of the optical conductivity σ_2 of Co_3O_4 (blue) plotted as a function of the photon energy at RT (**c**) and 7 K (**d**) along with the Kramers-Kronig of the σ_1 fit (gray).

Oscillator	7 K Energy (eV)	RT Energy (eV)	RT Ref.[24,26] Energy (eV)	Optical transitions assignment
1	0.85	0.82	0.83	on-site Co^{2+} d-d ($^4T_1$)
2	0.94	0.92	0.94	intersite d-d Co^{2+} 3d e → Co^{3+} 3d e_g
3	1.31	1.27	1.3 (RIXS)	intersite d-d Co^{2+} 3d t_2 → Co^{3+} 3d e_g
4 (A)	1.65	1.63	1.64	MH d-d Co^{3+} 3d t_{2g} → Co^{2+} 3d t_2
**Oscillator**	**7 K Energy (eV)**	**RT Energy (eV)**	**RT Ref.[24,26] Energy (eV)**	**Optical transitions assignment**
5 (B)	1.76	1.76	1.84	on-site d-d Co^{3+} ($^1T_{1g}$)<br>on-site d-d Co^{2+} ($^2E_1$)
6 (C)	2.73	2.71	2.62	CT O^{2-} 2p(I) → Co^{2+} 3d t_2
7 (D)	3.70	3.63	3.66	CT O^{2-} 2p(I) → Co^{3+} 3d e_g
8	4.76	4.68	4.69	CT O^{2-} 2p(II) → Co^{2+} 3d t_2
9	5.78	5.74	5.8	CT O^{2-} 2p(II) → Co^{3+} 3d e_g

Table S3. Oscillator central energies extracted from the gaussian deconvolution of the static optical conductivity at RT and 7 K, compared with the results reported in the literature[24,26]. The assignment of each optical transition is indicated in the last column. The capital letters used for the oscillators 4-7 in the first column of the table correspond to the labels used in the main text.

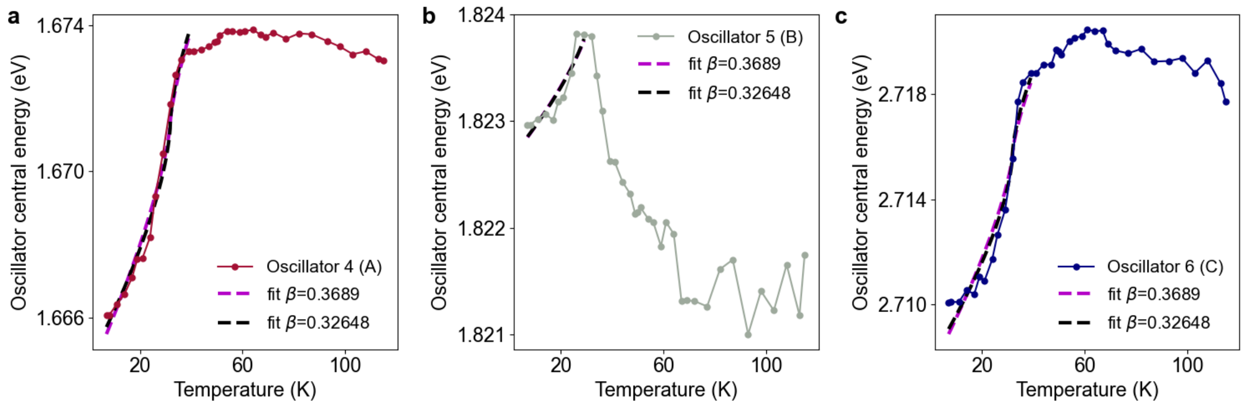

Figure S17. Oscillator central energies as a function of temperature and $(T - T_N)^{2\beta}$ fit (dashed lines) for: (**a**) oscillator 4 (MH intersite d-d transition), (**b**) oscillator 5 (on-site d-d transitions), (**c**) oscillator 6 (intersite p-d CT).

We describe the temperature evolution of the oscillators' central energies with the model presented in the main text: $E(T) = E_0 + a(E)(T - T_N)^{2\beta}$, where the critical exponent β reflects the 3D magnetic order of the system. The magnetic critical behaviour of spinel Co_3O_4 remains debated, with reports ranging from weak magnetic anisotropy consistent with a 3D Heisenberg description[29] (β=0.3689[30]) to strong axial anisotropy approaching the 3D Ising limit[31] (β=0.32648[32]). To account for this uncertainty, we performed global fits of the data in Figure S17a,c by fixing β to each of these limiting values. Both choices yield an equally good description of the experimental data and produce a consistent value of the global fit parameter $T_N \sim 32$ K, in excellent agreement with previous reports[29,33], confirming the validity of the magnetoelastic model. For clarity, the main text presents only the results obtained using the 3D Heisenberg model. We remark that an accurate determination of β is beyond the scope of the present work and would require dedicated magnetic measurements. We further note that the morphology of our thin film differs from that of bulk single crystals, as evidenced by the RHEED characterization (Figure S4), which may also influence the apparent critical behaviour.

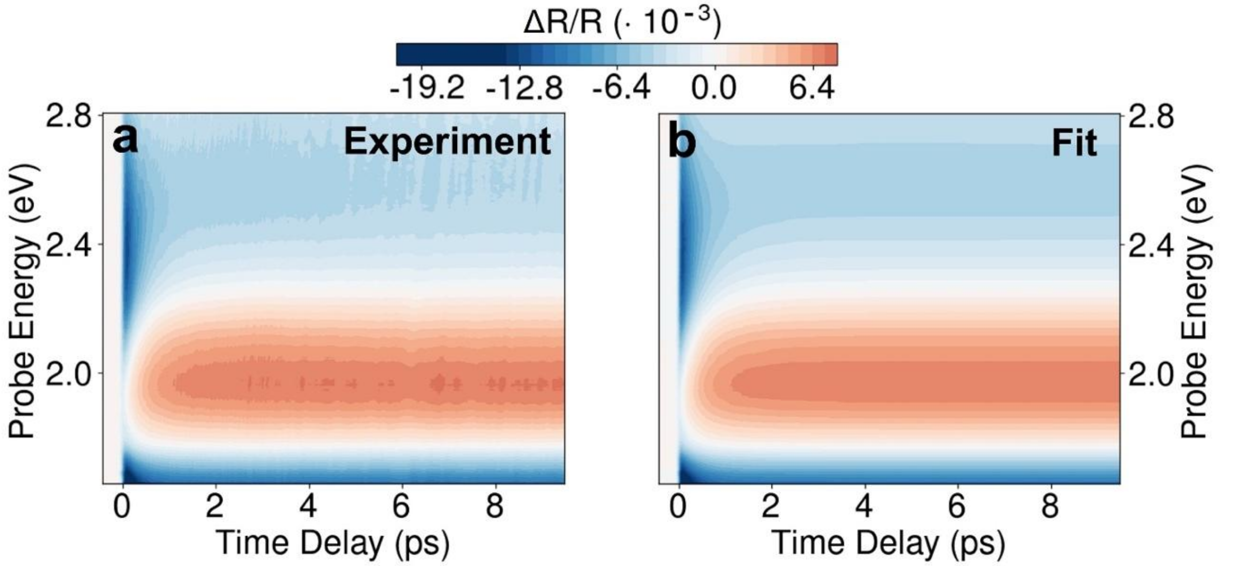

Figure S18. Transient reflectivity of Co_3O_4 at RT upon 3.10 eV excitation (photocarrier density: $3.1 \cdot 10^{20}$ cm^{-3}): (**a**) chirp-corrected ΔR/R data plotted as a function of time delay and probe energy. (**b**) GLA map obtained as best fit in OPTIMUS.

5. Co_3O_4 transient reflectivity

5.1 Transient reflectivity at RT

Figure S18a shows the chirp-corrected ΔR/R response of Co_3O_4 at RT upon 3.10 eV excitation (photocarrier density of $3.1 \cdot 10^{20}$ cm^{-3}) as a function of time delay and probe energy. Figure S18b reports the corresponding maps obtained through a global lifetime analysis (GLA) using the OPTIMUS software[7], following the procedure described in Section 2.2.

The time constants of the GLA multiexponential fit shown in Figure S18 are listed in Table S4 together with those obtained at RT upon 1.55 eV photoexcitation (photocarrier density of $1.5 \cdot 10^{20}$ cm^{-3}). The results in Table S4 show the presence of an additional ultrafast time component upon 3.10 eV photoexcitation with respect to the 1.55 eV pump photon energy, while the other time constants are in good agreement with each other and with the values reported in the literature[34,35]. Figure S19a,b shows the comparison between the ΔR/R time traces of Co_3O_4 at 1.65 eV and 2.4 eV probe photon energies, normalized by the plateau intensity region (~5 ps).

The transients are in very good agreement, except for the first few hundreds of fs, when the traces upon 3.10 eV photoexcitation undergo the additional decay process retrieved by the GLA. The transient energy spectra are reported in Figure S19c, showing similar shapes. Both traces are characterized by a negative signal in the 1.65-1.80 eV range, a positive one in the 1.80-2.30 eV range, and a negative one in the 2.30-2.70 eV range.

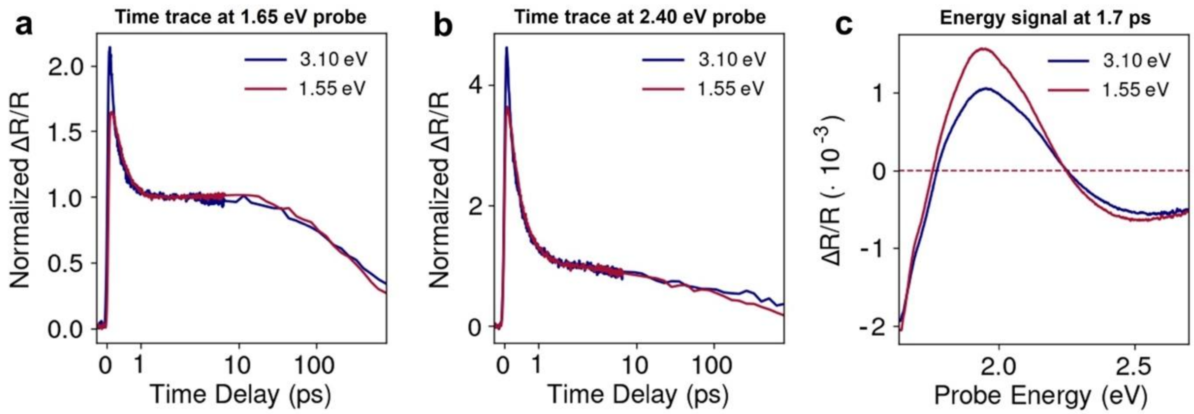

Figure S19. Comparison between ΔR/R time traces upon 1.55 eV and 3.10 eV photoexcitation at (**a**) 1.65 eV and (**b**) 2.4 eV probe energies, normalized by the intensity plateau at 5 ps. The x-axis is in log_{10} scale. (**c**) Comparison between ΔR/R energy traces upon 1.55 eV and 3.10 eV pump at 1.7 ps time delay.

Experimental conditions	τ_1 (ps)	τ_2 (ps)	τ_3 (ps)	τ_4 (ps)	τ_5 (ps)
1.55 eV pump, RT	0.41	5.9	204	Offset	--
3.10 eV pump, RT	0.19	0.55	8.00	229	Offset

Table S4. Time constants obtained from the GLA of the transient reflectivity maps measured at RT upon 1.55 eV and 3.10 eV photoexcitation.

RT	1.55 eV pump	3.10 eV pump
Incident fluence ($mJ\cdot cm^{-2}$)	0.2 – 9.6	0.6 – 6.8
Excitation density (cm^{-3})	$3.0\cdot10^{19}$ – $1.9\cdot10^{21}$	$3.1\cdot10^{20}$ – $3.6\cdot10^{21}$
**4.2 K**	**1.55 eV pump**	**3.10 eV pump**
Incident fluence ($mJ\cdot cm^{-2}$)	0.2 – 0.8	0.6 – 3.0
Excitation density (cm^{-3})	$3.4\cdot10^{19}$ – $1.0\cdot10^{20}$	$2.7\cdot10^{20}$ – $1.2\cdot10^{21}$

Table S5. Linear response of the Co_3O_4 transient reflectivity signal as a function of the excitation fluence at RT and 4.2 K.

5.2 Transient reflectivity as a function of pump fluence and temperature

The transient reflectivity response of spinel Co_3O_4 was characterized as a function of pump fluence at both RT and 4.2 K. The absorbed pump fluence was normalized by the photon energy $h\nu$ and the penetration depth d, and expressed in terms of the charge carriers' excitation density n_e as:

$$n_e = \frac{F}{h\nu \cdot d}[1 - R(\theta)] \quad \text{(S3)}$$

where θ is the angle of incidence between the pump beam and the sample surface.

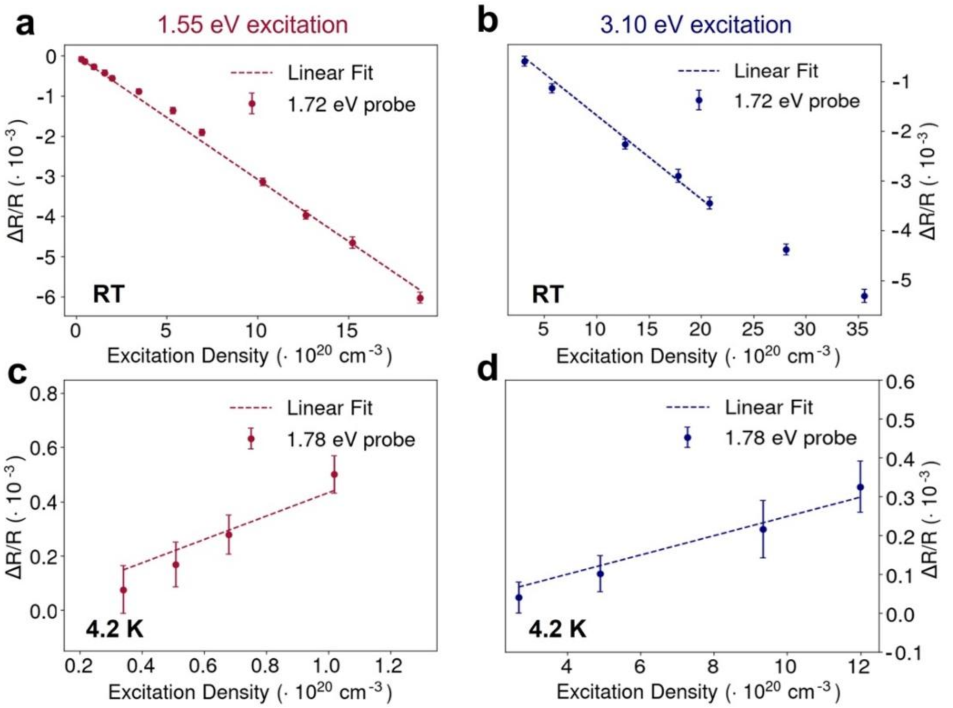

Figure S20. Co_3O_4 fluence dependence of the oscillator 5 measured at 1.7 ps, for (**a**) 1.55 eV pump at RT, (**b**) 3.10 eV pump at RT, (**c**) 1.55 eV pump at 4.2 K and (**d**) 3.10 eV pump at 4.2 K. The linear fits are indicated as dashed lines.

The photoresponse was investigated for incident fluences in the ranges 0.2-9.6 mJ·cm^{-2} (0.2-6.0 mJ·cm^{-2}) for the 1.55 eV pump at RT (4.2 K), and 0.6-6.8 mJ·cm^{-2} (0.6-6.0 mJ·cm^{-2}) for the 3.10 eV pump photon energy at RT (4.2 K). Figure S20 shows the fluence dependence upon 1.55 eV (panel a: RT; panel c: 4.2 K) and 3.10 eV (panel b: RT; panel d: 4.2 K) photoexcitation, measured at 1.7 ps at the probe photon energy corresponding to the peak energy of the oscillator 5. The range of linearity (highlighted by the dashed red line) is significantly more extended for the measurements at RT than at 4.2 K. All pump-probe measurements reported in the manuscript were performed within the linear fluence regime except for the highest fluence at 4.2 K and 3.10 eV excitation photon energy (4.7 mJ·cm^{-2}, corresponding to 20.9·10^{20} cm^{-3}). The experimental conditions corresponding to the linear response are summarized in Table S4.

Figure S21 shows the normalized transient amplitudes of the oscillator 5 (on-site d-d transition labeled as B in the main text) for both 1.55 eV (Figure S21a) and 3.10 eV (Figure S21b) excitation at RT and 4.2 K. The traces were normalized to the first maximum after the rise time at about 100 fs and were obtained by exciting the sample within the linear regime, with photocarrier densities of 1.4·10^{20} cm^{-3} for 1.55 eV excitation and of 4.9·10^{20} cm^{-3} for 3.10 eV excitation. At both temperatures, the ultrafast response is similar up to 8 ps, but at longer time scales it changes significantly: at 4.2 K a delayed rise is detected in the 10-100 ps time range for both excitation photon energies.

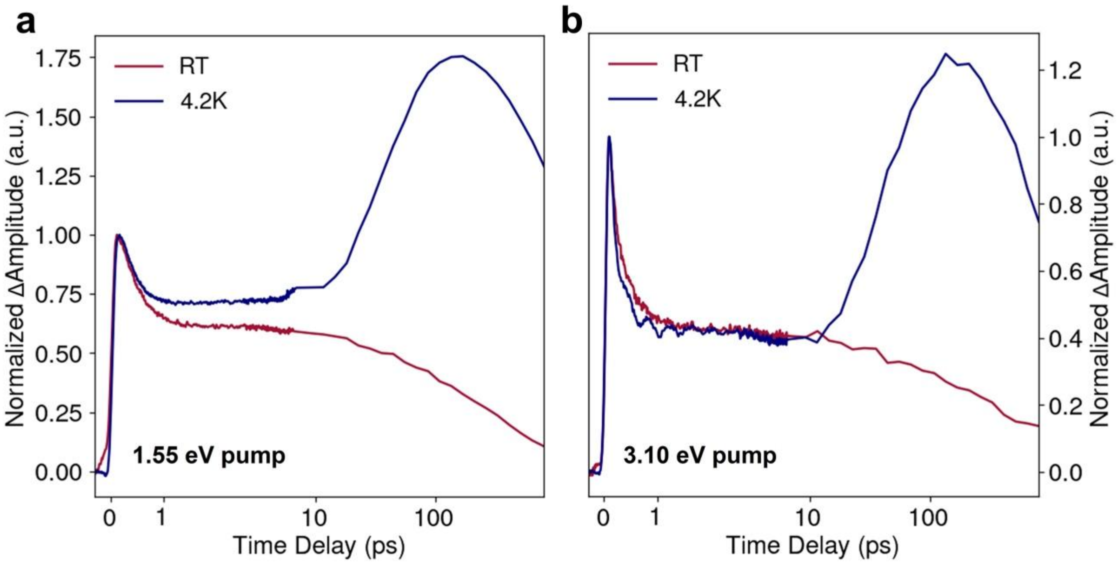

Figure S21. Comparison between RT (above T_N) and 4.2 K (below T_N) transient amplitude variations of the on-site d-d oscillator 5 (labeled as B in the main text) upon (**a**) 1.55 eV and (**b**) 3.10 eV pump photon energies. The traces are normalized to the first maximum after the rise time at ~100 fs and the x-axis is in $\log_{10}$ scale.

We attribute this feature to a delayed melting of the antiferromagnetic (AFM) order due to the temperature rise of the system above T_N, similarly to $TbMnO_3$[36]. This assignment is corroborated by our estimates of laser heating effect on the sample. We computed the maximum transient lattice temperature that is reached in the Co_3O_4 film upon photodoping for the two excitation photon energies, 1.55 eV and 3.10 eV, using the following expression:

$$Q = \int_{T_i}^{T_f} m \cdot C(T) dT \quad \text{(S4)}$$

where Q is the absorbed heat from a single laser pulse, m is the illuminated mass, $C(T)$ is the temperature-dependent specific heat, T_i = 4.5 K is the initial equilibrium temperature, and T_f is the final temperature. The mass m is calculated through the material density ρ = 6.11 g/cm^3, and the illuminated sample volume V. V is calculated by multiplying the beam spot size and the penetration depth, which is calculated from the optical conductivity data. We extracted the temperature dependence of the heat capacity from a previous report[33]. For 1.55 eV pump photon energy, the minimum excitation density used in the experiments yields $T_{f,1.55eV,min}$ = 30.1 K, whereas at the maximum excitation density we obtain $T_{f,1.55eV,max}$ = 63.7 K. For 3.10 eV pump photon energy, the minimum excitation density yields $T_{f,3.10eV,min}$ = 58.0 K, and the maximum excitation density gives $T_{f,3.10eV,max}$ = 120.1 K. Except for the 1.55 eV lowest fluence scenario, the estimated maximum temperatures are above the Néel temperature of Co_3O_4, in agreement with the transient melting of the AFM order observed in Figure S21a,b at time scales longer than 10 ps. At shorter time scales, instead, no experimental evidence of transient lattice temperature increase is observed.

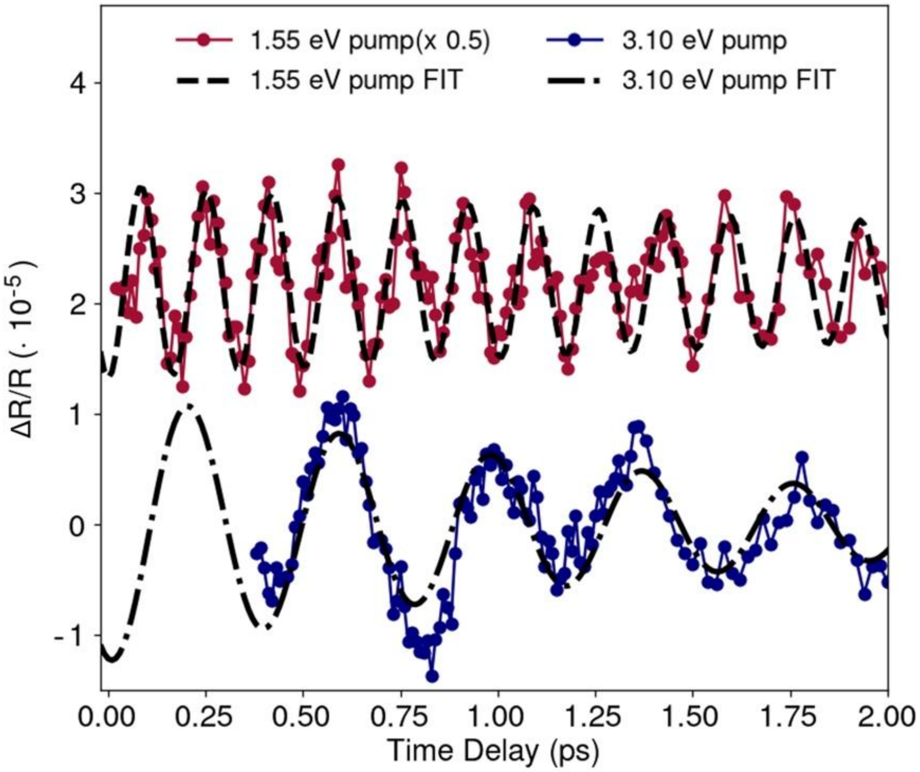

Figure S22. Transient reflectivity coherent response of Co_3O_4 upon 1.55 eV photoexcitation ($1.4 \cdot 10^{20}$ cm^{-3}) at 70 K (red dots) and 3.10 eV photoexcitation ($4.9 \cdot 10^{20}$ cm^{-3}) at 150 K (blue dots). Both oscillations are fitted with damped cosine functions (black lines) and the fit is extended to zero time delay to extract the oscillation phase. The region near time-zero is omitted due to the presence of a coherent artifact, and a vertical offset was introduced for clarity.

5.3 Coherent response

5.3.1 Phase and damping time

We determined the phase and damping time of the coherent oscillations reported in the main text (Figure 2d) by fitting them with a damped cosine function. The results are shown in Figure S22, corresponding to a phase $\phi = 0.02 \pm 0.08$ rad and a damping time $t_D = 4.2 \pm 2.0$ ps for the mode at 24.3 meV, obtained upon 1.55 eV photoexcitation at 70 K, and $\phi = 0.15 \pm 0.10$ rad and $t_D = 1.5 \pm 0.3$ ps for the 10.2 meV mode, obtained upon 3.10 eV photoexcitation at 150 K. We note that the relatively high error on t_D upon 1.55 eV excitation is due to the limited damping of the coherent oscillations in the probed time window, which is limited to the first 2 ps of the dynamics. As discussed in the main text, both collective excitations are ascribed to coherent phonons, either present (T_{2g} phonon at 24.3 meV) or absent (10.2 meV mode) in the spontaneous Raman spectrum of the system. A unified framework has been proposed to describe the mechanism driving coherent phonons in terms of stimulated Raman scattering tensors[37]. This harmonizes what was previously distinguished in terms of impulsive stimulated Raman scattering (ISRS) or displacive excitation of coherent phonons (DECP)[38].

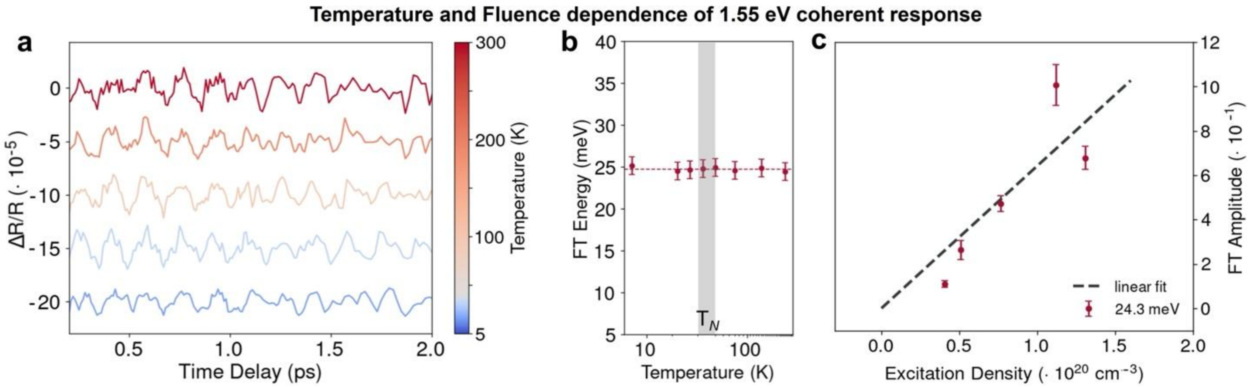

Figure S23. Transient reflectivity coherent response of Co_3O_4 as a function of temperature (4.2 K, 22 K, 40 K, 70 K, RT) upon 1.55 eV pumping at a constant excitation density of $1.4 \cdot 10^{20}$ cm^{-3} (0.8 $mJ \cdot cm^{-2}$ incident fluence), spectrally averaged over the 1.65-1.75 eV probe photon energy range. (**a**) Coherent oscillations as a function of the time delay. (**b**) Oscillation energy as a function of temperature in linear scale up to 50 K and $\log_{10}$ scale up to RT. The range of temperature covering T_N[29,33] is highlighted with a shaded light gray area. The red dashed line corresponds to the mean value across the temperature. The error bars of the FT energy correspond to the uncertainty defined by the time window of the FT. (**c**) Amplitude of the FT maximum as a function of the excitation fluence for 1.55 eV pump and linear fit (dashed gray line). The error bars of the amplitude correspond to the standard error obtained upon fitting the FT spectrum with a Lorentzian function.

5.3.2 1.55 eV photoexcitation as a function of temperature and fluence

Figure S23a shows the coherent response of Co_3O_4 upon 1.55 eV photoexcitation as a function of temperature. The traces were obtained by subtracting the incoherent background given by the GLA multiexponential fit and a polynomial function from the transient reflectivity data and by spectrally averaging in the 1.65-1.75 eV probe energy region. The oscillations have a period of 167±20 fs, corresponding to an energy of 24.3±2.0 meV, and a damping time of $t_D > 2$ ps. Figure S23b shows the corresponding FT as a function of temperature. Zero-padding and Kaiser-Bessel windowing with $\beta = 1$[8] were applied prior to FT. The central energy of the oscillations remains constant over the entire temperature range from 4.2 K to RT. Figure S23c shows the 4.2 K photocarrier density dependence of the 24.3 meV FT amplitude fitted with linear functions up to $1.0 \cdot 10^{20}$ cm^{-3}. The reported linear behaviour is compatible with a coherent phonon excitation[38], and it is systematically observed in the transient reflectivity signals for both RT and 4.2 K.

5.3.3 3.10 eV photoexcitation as a function of temperature and fluence

Figure S24a shows the coherent response of the Co_3O_4 upon 3.10 eV photoexcitation as a function of temperature. The traces were obtained following the same procedure described for the 1.55 eV photoexcitation, and spectrally averaging over the 2.20-2.50 eV probe photon range. We obtained an oscillation period of 405±20 fs, which correspond to an energy of 10.2±1.5 meV, and a damping time <2 ps. The central energy of the FT exhibits no change across the temperature range from 4.2 K to RT (Figure S24b).

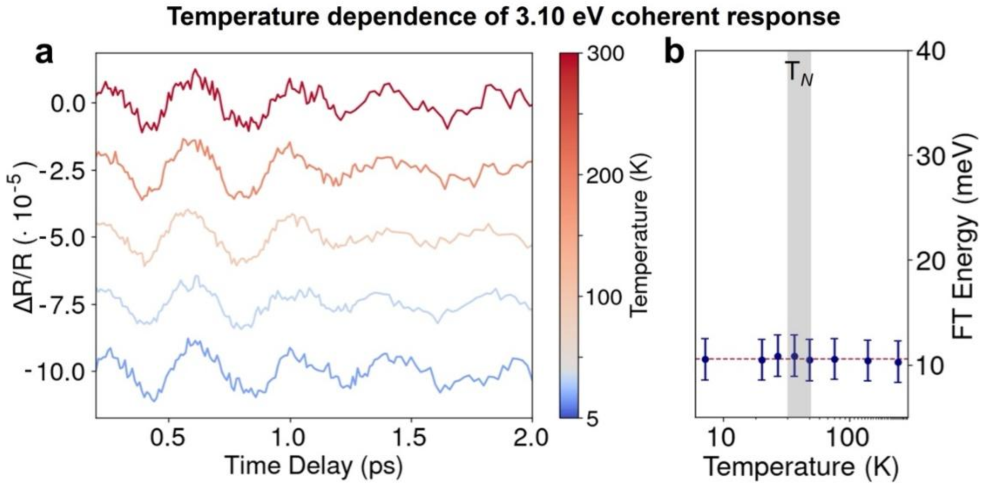

Figure S24. Transient reflectivity coherent response of Co_3O_4 as a function of the temperature (4.2 K, 22 K, 40 K, 70 K, RT) upon 3.10 eV excitation at a constant excitation density of $4.9 \cdot 10^{20}$ cm^{-3} (1.1 $mJ \cdot cm^{-2}$ incident fluence), spectrally averaged in the 2.20-2.50 eV probe energy range. (**a**) Coherent oscillations as a function of the time delay. (**b**) Oscillation frequency as a function of the temperature in linear scale up to 50 K and $\log_{10}$ scale up to RT. The range of temperature covering T_N is highlighted with a shaded light gray area. The red dashed line corresponds to the mean value across the temperature. The error bars correspond to the energy uncertainty defined by the time window of the FT.

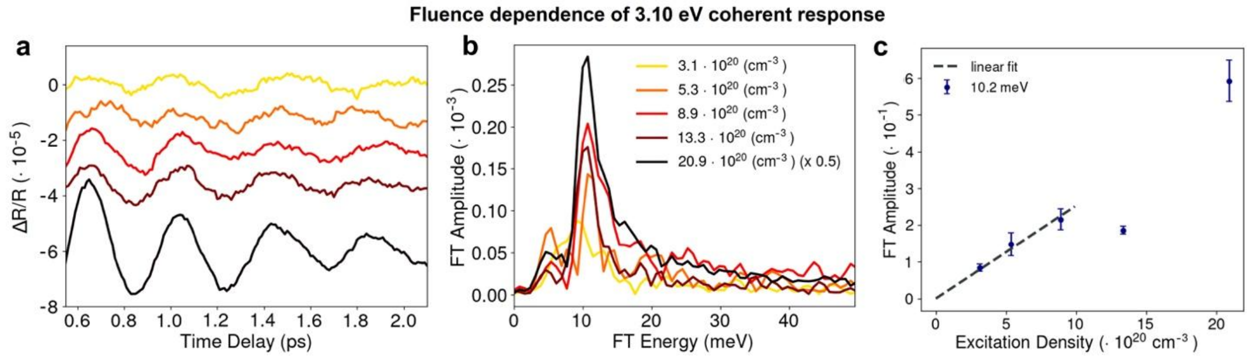

Figure S25. Transient reflectivity coherent response of Co_3O_4 as a function of photoexcitation density upon 3.10 eV excitation, 4.2 K, spectrally averaging the probe in the photon energy range 2.30-2.70 eV. (**a**) Coherent oscillations as a function of the time delay. (**b**) FT spectra of the traces reported in panel (**a**). (**c**) Amplitude of the FT maximum as a function of the excitation fluence for 3.10 eV pump and linear fit (dashed gray line). The error bars represent the standard error in the amplitude determined from Lorentzian fitting of the FT traces.

As discussed in the main text, this collective excitation is ascribed to a phonon mode that has no correspondence to the zone center of the $Fd\overline{3}m$ space group. Other possible assignments of this mode are discussed and excluded in Section 6. The fluence dependence of the coherent response upon 3.10 eV photoexcitation at 4.2 K is shown in Figure S25.

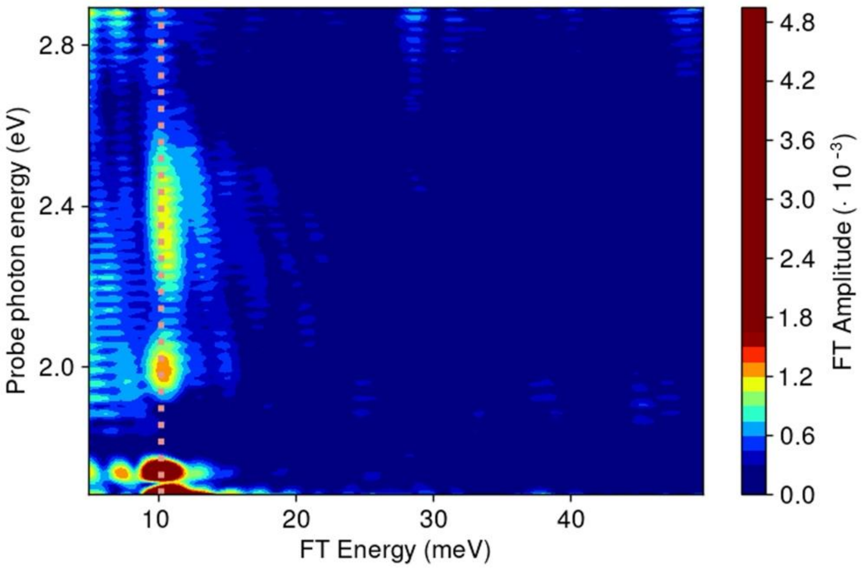

Figure S26. FT amplitude map of the 10.2 meV oscillation obtained upon 3.10 eV photoexcitation ($2.1 \cdot 10^{21}$ cm^{-3} photocarrier density) and 1.7-2.90 eV probe. The dotted line is set at 10.2 meV constant frequency along the whole probe energy range.

The traces as a function of time (Figure S25a) were obtained following the same procedure as in Section 5.3.2 and spectrally averaging the probe photon energy range in the interval 2.30-2.70 eV. Their FT is reported in Figure S25b and their amplitude at 10.2 meV is shown as a function of the photocarrier density in Figure S25c. The linear behaviour is compatible with the coherent phonon excitation process[38].

6. Assignment of the 10.2 meV collective mode

6.1 Acoustic phonons

In this paragraph, the possibility that the coherently evolving collective mode arises from acoustic phonons is first evaluated and ruled out. A photogenerated propagating acoustic phonon should satisfy two conditions: (i) the mode frequency should exhibit a linear dispersion with the probe energy due to Brillouin scattering; (ii) the calculated sound velocity of the acoustic phonon mode should match the typical sound velocity of Co_3O_4 of $v_s \sim 6$ km/s, as predicted by DFT calculations and experimental measurements[22].

Our DFT calculations predict the presence of an acoustic branch crossing an energy of 10.2 meV between the Γ and L, X, and K points of the Brillouin zone (Figure S15). Figure S26 reports the FT amplitude map of the coherent response of the system as a function of the FT energy and probe photon energy upon 3.10 eV photoexcitation (photocarrier density of $2.1 \cdot 10^{21}$ cm^{-3}) at 4.2 K. The oscillation frequency shows no measurable dispersion within the 2.20-2.60 eV probe energy range, indicating that it does not originate from propagating coherent acoustic phonons.

Phonon mode	DFT calculations[39], Energy (meV)	Neutron diffraction[40], Energy (meV)	IR spectroscopy[41], Energy (meV)
TO	42.4	43.4	42.6
LO	65.1	65.2	67.4

Table S6. T_{1u} longitudinal and transverse optical mode frequencies in CoO.

Furthermore, we estimated the corresponding sound velocity if the mode were a propagating coherent acoustic phonon. We used the relation[42]:

$$v_s = \frac{4d}{\tau_0} \quad \text{(S5)}$$

where d is the thickness of the film and τ_0 is the period of the oscillation. Using d = 27 nm and τ_0 = 405 fs, we obtain $v_s \sim 265$ km/s, an unphysically large velocity for propagating sound waves. We also calculated the Young's modulus starting from the estimated v_s through the following formula:

$$Y = v_s^2\, \rho \quad \text{(S6)}$$

where ρ is the system's density (6 g/cm^3). We obtain $Y \sim 4{\cdot}10^5$ GPa, which is two orders of magnitude higher than diamond (1050 GPa). These calculations demonstrate that the oscillation at 10.2 meV is not compatible with a propagating acoustic phonon.

6.2 CoO phonons

Wdowik *et al.* reported the phonon dispersion curves of CoO obtained from *ab initio* calculations and inelastic neutron scattering at 110 K[39]. This system has a rocksalt structure ($Fm\bar{3}m$ space group) with a single optical phonon at the Γ point, which has T_{1u} symmetry and is infrared-active. It splits into transverse (TO) and longitudinal (LO) optical components, whose energies are listed in Table S6. The lowest mode at the Brillouin zone center has an energy of ∼43 meV, *i.e.*, about four times higher than the 10.2 meV coherent oscillation observed in our experiment. Moreover, its infrared activity is incompatible with a Raman generation mechanism. The absence of any of the above in our experimental data further corroborates our samples quality and chemical purity.

6.3 Sapphire substrate phonons

Our sample consists of a 27 nm Co_3O_4 thin film epitaxially grown along the $[0001]$ direction on a sapphire (α-Al_2O_3) substrate by pulsed laser deposition[1]. Sapphire crystallizes in a trigonal structure with point group $\bar{3}m$ (D_{3d}) and space group $R\bar{3}c$ (D_{3d}^6). Group theory predicts 18 phonon modes at the Γ point with the following irreducible representation[43]:

$$\Gamma = 2A_{1g} + 2A_{1u} + 3A_{2g} + 3A_{2u} + 5E_g + 5E_u \quad \text{(S7)}$$

Energy (meV)	Raman, infrared, inactive mode	Symmetry
37.3	N	A_{2g}
46.9	R	E_g
48.0	I	E_u
49.6	I	A_{2u}
51.8	R	A_{1g}
53.6	R	E_g
55.1	I	E_u
55.9	R	E_g
66.5	N	A_{2g}
70.7	I	E_u
71.7	R	E_g
72.4	I	A_{2u}
73.7	N	A_{1u}
79.0	I	E_u
80.0	R	A_{1g}
85.3	N	A_{1u}
92.8	N	A_{2g}
93.1	R	E_g

Table S7. Raman (R), infrared (I) and inactive (N) phonon mode energies of sapphire reported in the literature[43]. The symmetry of each mode is reported is in the third column.

The two A_{1g} and the five E_g phonons are Raman-active, two A_{2u} phonons and four E_u phonons are infrared-active, one of the A_{2u} and E_u phonons are acoustic modes, and the A_{1u} and A_{2g} modes are inactive. The phonons frequencies and symmetries at the Γ point are summarized in Table S7, based on literature results[43]. The lowest optical phonon, which is inactive and has A_{2g} symmetry, lies of 37.3 meV, far above the 10.2 meV oscillation observed in transient reflectivity upon 3.10 eV photoexcitation.

6.4 Single magnons and bi-magnon excitations

Single magnons and bi-magnon excitations may coherently modulate the transient reflectivity response of a magnetic solid[44,45]. Single magnons exist only in the AFM phase and its proximity, *i.e.*, in spinel Co_3O_4 around and below the $T_N \sim$ 30–40 K[29,33]. Our data show that the oscillations at 10.2 meV are present even at RT and that the FT central energy does not change with temperature (see Figure S24b). Therefore, the collective mode cannot be ascribed to a single magnon excitation.

Bi-magnons, instead, are linked to short-range magnetic correlations and typically persist well above T_N. These excitations are Raman-active and are therefore detected in spontaneous Raman scattering experiments[46–49]. They often exhibit a continuum-like response that translates into overdamped oscillations in the time-domain. Furthermore, their central energy, intensity and

linewidth are sensitive to the temperature of the system, as observed for bi-magnons in MnTe[46,47]. Given the long damping time of the 10.2 meV oscillation (t_D = 1.5±0.3 ps), its temperature independence and its absence in our spontaneous Raman scattering measurements, we exclude the bi-magnon origin of this low-energy collective mode.

6.5 Pump-induced structural transition

Impulsive photoexcitation of spinel Co_3O_4 could, in principle, induce an ultrafast structural phase transition toward another structure characterized by a phonon mode at 10.2 meV at the Γ point, which would coherently modulate the transient reflectivity signal. The phases that are directly related to the original cubic $Fd\overline{3}m$ space group are the orthorhombic $Fddd$, and the monoclinic $C2/m$ and $P2_1/c$ ones, as observed in the Co_3O_4 phase diagram at RT[50]. We thus performed DFT calculations with the GGA approximation to compute the phonon dispersion of spinel Co_3O_4 in the orthorhombic $Fddd$ and monoclinic $P2_1/c$ phases, respectively shown in Figure S27 and Figure S28. The corresponding phonon energies at the Γ point of the Brillouin zone are reported in Table S8 and Table S9. We note that DFT simulations of the $C2/m$ monoclinic structure were not performed due to the lack of lattice parameters describing this phase and to the difficulty of convergence of the calculations.

For the $Fddd$ space group, 39 phonons were obtained at the Γ point, with the lowest phonon energy around 24 meV, *i.e.*, well above the 10.2 meV of the coherent oscillation observed in transient reflectivity. More pronounced changes are observed in the $P2_1/c$ phase, which contains two classes of octahedra, Co^{2+} and Co^{3+}, interconnected by shared edges forming an open layered structure along the $[001]$ direction[50]. This is a direct consequence of the increased coordination number of the Co^{2+} sites from 4 in $Fd\overline{3}m$ to 6 in $P2_1/c$ lattice. As a result, the cation–cation bond distances become nearly the same for all Co centres: Co^{2+}–Co^{2+}=2.73 Å, Co^{2+}–Co^{3+}=2.68 Å and Co^{3+}–Co^{3+}=2.73 Å. Furthermore, the Co^{2+} and Co^{3+} octahedra are highly distorted, with average Co^{2+}–O^{2-} and Co^{3+}–O^{2-} bond distances of 1.89 Å and 1.92 Å, respectively. In this phase, the lowest optical phonon energy at the Γ point is approximately 15 meV. Even though the collective mode induced by the 3.10 eV pump pulse is close in energy to this optical phonon, we exclude the photoinduced phase transition scenario for the following reasons: (i) the lowest-energy mode is infrared-active and could be excited only upon centrosymmetry breaking of the $Fd\overline{3}m$ and $P2_1/c$ unit cells; (ii) the structural modifications required to reach the $P2_1/c$ space group involve significant changes of the lattice structure, including an increase in coordination number of the Co^{2+} tetrahedral sites from 4 to 6, which is unlikely given that the 3.10 eV pump process predominantly excites Co^{3+} octahedral centers; (iii) a global lattice symmetry change would modify other experimental observables, such as the optical d-d transitions, which are not observed in our transient reflectivity measurements.

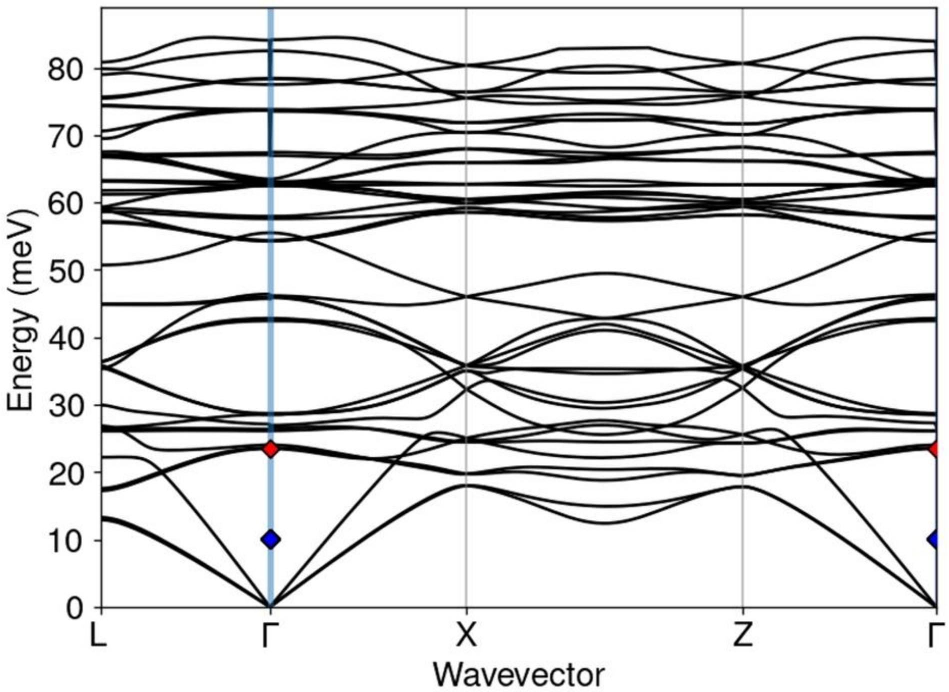

Figure S27. Phonon dispersion in the orthorhombic $Fddd$ space group of Co_3O_4 calculated through DFT. The blue and red diamonds mark the energy positions of the 10.2 meV and 24.3 meV phonons.

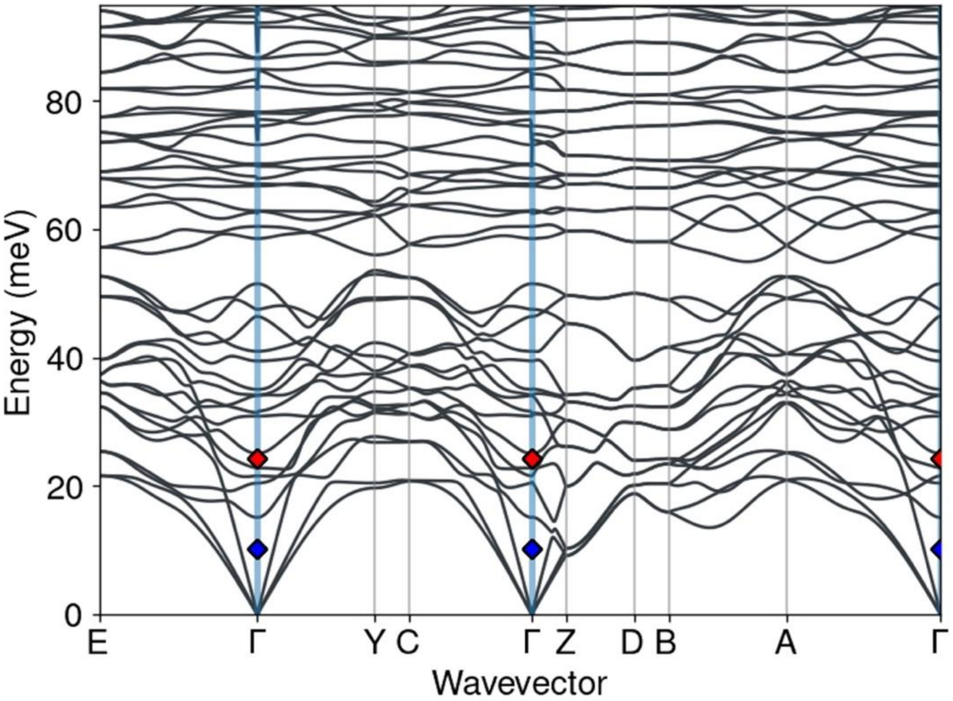

Figure S28. Phonon dispersion in the monoclinic $P2_1/c$ structure of Co_3O_4 calculated through DFT. The blue and red diamonds mark the energy positions of the 10.2 meV and 24.3 meV phonons.

Energy (meV)	Raman (R), infrared (I), Inactive (N) mode	Symmetry
23.5	R	B_{3g}
23.6	R	B_{2g}
24.1	R	B_{1g}
26.1	I	B_{3u}
26.1	I	B_{2u}
26.6	I	B_{1u}
28.5	I	B_{3u}
28.6	I	B_{2u}
28.7	I	B_{1u}
42.5	N	A_u
42.8	N	A_u
45.7	I	B_{2u}
45.9	I	B_{3u}
46.1	I	B_{1u}
54.3	R	B_{1g}
54.3	N	A_u
54.4	R	B_{2g}
55.5	R	B_{3g}
57.6	R	A_g
57.9	R	A_g
62.5	I	B_{3u}
62.5	I	B_{2u}
62.7	N	A_u
62.9	I	B_{1u}
63.1	N	A_u
63.1	R	B_{1g}
63.1	R	B_{3g}
63.5	R	B_{2g}
67.0	I	B_{1u}
67.2	I	B_{3u}
67.5	I	B_{2u}
73.7	R	B_{3g}
73.7	R	B_{1g}
73.8	R	B_{2g}
77.5	I	B_{3u}
78.3	I	B_{2u}
78.3	I	B_{1u}
78.5	N	A_u
82.5	R	A_g

Table S8. Raman (R), infrared (I), and inactive (N) phonon mode energies obtained from DFT calculations for the $Fddd$ structure of spinel Co_3O_4. The symmetry of each mode is reported in the third column.

Energy (meV)	Raman (R), IR (I), Inactive (N) mode	Symmetry
15.1	I	A_u
20.4	I	A_u
21.6	R	A_g
22.8	I	B_u
25.1	R	A_g
30.9	R	B_g
31.4	I	B_u
34.1	I	A_u
34.2	I	A_u
35.1	I	B_u
39.5	R	B_g
41.0	R	B_g
46.4	R	A_g
47.6	I	B_u
51.5	I	A_u
58.6	R	B_g
60.5	R	A_g
62.5	I	B_u
62.9	I	A_u
66.8	R	A_g
67.1	R	B_g
67.9	I	B_u
69.8	I	A_u
70.2	R	A_g
73.2	I	B_u
73.9	I	A_u
76.1	R	B_g
78.1	R	B_g
78.3	R	A_g
81.9	I	A_u
82.2	I	B_u
85.0	I	A_u
86.6	R	A_g
86.7	I	B_u
87.7	R	B_g
92.2	I	B_u
93.4	R	A_g
94.2	I	A_u
94.3	R	B_g

Table S9. Raman (R), infrared (I), and inactive (N) phonon mode energies obtained from DFT calculations for the $P2_1/c$ structure of spinel Co_3O_4. The symmetry of each mode is reported in the third column.

References

(1) Hubler, G. K. Pulsed Laser Deposition. *MRS Bull.* **1992**, *17* (2), 26–29. https://doi.org/10.1557/S0883769400040586.

(2) Baumgartel, H. EXAFS, SEXAFS, XANES: X-Ray Absorption - Principles, Applications, Techniques of EXAFS, SEXAFS and XANES. Von D. Koningsberger Und R. Prins. John Wiley & Sons Ltd., Chichester 1988. 673 S., Abb., Tab., Formeln. ISBN 0-471-87547-3. *Nachrichten Aus Chem. Tech. Lab.* **1988**, *36* (6), 650–650. https://doi.org/10.1002/nadc.19880360617.

(3) Bordage, A.; Trannoy, V.; Proux, O.; Vitoux, H.; Moulin, R.; Bleuzen, A. In Situ Site-Selective Transition Metal K-Edge XAS: A Powerful Probe of the Transformation of Mixed-Valence Compounds. *Phys. Chem. Chem. Phys.* **2015**, *17* (26), 17260–17265. https://doi.org/10.1039/C5CP02591E.

(4) Mancini, G. F.; Mansart, B.; Pagano, S.; Van Der Geer, B.; De Loos, M.; Carbone, F. Design and Implementation of a Flexible Beamline for Fs Electron Diffraction Experiments. *Nucl. Instrum. Methods Phys. Res. Sect. Accel. Spectrometers Detect. Assoc. Equip.* **2012**, *691*, 113–122. https://doi.org/10.1016/j.nima.2012.06.057.

(5) CrystalMaker® CrystalMaker Software Ltd. www.crystalmaker.com.

(6) Biesinger, M. C.; Payne, B. P.; Grosvenor, A. P.; Lau, L. W. M.; Gerson, A. R.; Smart, R. St. C. Resolving Surface Chemical States in XPS Analysis of First Row Transition Metals, Oxides and Hydroxides: Cr, Mn, Fe, Co and Ni. *Appl. Surf. Sci.* **2011**, *257* (7), 2717–2730. https://doi.org/10.1016/j.apsusc.2010.10.051.

(7) Slavov, C.; Hartmann, H.; Wachtveitl, J. Implementation and Evaluation of Data Analysis Strategies for Time-Resolved Optical Spectroscopy. *Anal. Chem.* **2015**, *87* (4), 2328–2336. https://doi.org/10.1021/ac504348h.

(8) Harris, F. J. On the Use of Windows for Harmonic Analysis with the Discrete Fourier Transform. *Proc. IEEE* **1978**, *66* (1), 51–83. https://doi.org/10.1109/PROC.1978.10837.

(9) Kresse, G.; Furthmüller, J. Efficient Iterative Schemes for *Ab Initio* Total-Energy Calculations Using a Plane-Wave Basis Set. *Phys. Rev. B* **1996**, *54* (16), 11169–11186. https://doi.org/10.1103/PhysRevB.54.11169.

(10) Dudarev, S. L.; Botton, G. A.; Savrasov, S. Y.; Humphreys, C. J.; Sutton, A. P. Electron-Energy-Loss Spectra and the Structural Stability of Nickel Oxide: An LSDA+U Study. *Phys. Rev. B* **1998**, *57* (3), 1505–1509. https://doi.org/10.1103/PhysRevB.57.1505.

(11) Chen, J.; Wu, X.; Selloni, A. Electronic Structure and Bonding Properties of Cobalt Oxide in the Spinel Structure. *Phys. Rev. B* **2011**, *83* (24), 245204. https://doi.org/10.1103/PhysRevB.83.245204.

(12) Smith, W. L.; Hobson, A. D. The Structure of Cobalt Oxide, Co_3O_4. *Acta Crystallogr. B* **1973**, *29* (2), 362–363. https://doi.org/10.1107/S0567740873002505.

(13) Parlinski, K.; Li, Z. Q.; Kawazoe, Y. First-Principles Determination of the Soft Mode in Cubic ZrO_2. *Phys. Rev. Lett.* **1997**, *78* (21), 4063–4066. https://doi.org/10.1103/PhysRevLett.78.4063.

(14) Parlinski, K. PHONON Software. *Comput. Mater.* **2013**.

(15) Togo, A.; Chaput, L.; Tadano, T.; Tanaka, I. Implementation Strategies in Phonopy and Phono3py. *J. Phys. Condens. Matter* **2023**, *35* (35), 353001. https://doi.org/10.1088/1361-648X/acd831.

(16) White, W. B.; DeAngelis, B. A. Interpretation of the Vibrational Spectra of Spinels. *Spectrochim. Acta Part Mol. Spectrosc.* **1967**, *23* (4), 985–995. https://doi.org/10.1016/0584-8539(67)80023-0.

(17) Malone, E. M.; Petitto, S. C.; Langell, M. A. Fuchs–Kliewer Phonon Spectrum of Co_3O_4(110) Single Crystal Surfaces by High Resolution Electron Energy Loss Spectroscopy. *Solid State Commun.* **2004**, *130* (9), 571–575. https://doi.org/10.1016/j.ssc.2004.03.040.

(18) Hadjiev, V. G.; Iliev, M. N.; Vergilov, I. V. The Raman Spectra of Co_3O_4. *J. Phys. C Solid State Phys.* **1988**, *21* (7), L199–L201. https://doi.org/10.1088/0022-3719/21/7/007.

(19) Lei, Z.; Chen, X.; Wang, J.; Huang, Y.; Du, F.; Yan, Z. Guite, the Spinel-Structured $Co^{2+}Co^{3+}{}_2O_4$, a New Mineral from the Sicomines Copper–Cobalt Mine, Democratic Republic of Congo. *Mineral. Mag.* **2022**, *86* (2), 346–353. https://doi.org/10.1180/mgm.2022.27.

(20) Mock, A.; Korlacki, R.; Briley, C.; Sekora, D.; Hofmann, T.; Wilson, P.; Sinitskii, A.; Schubert, E.; Schubert, M. Anisotropy, Band-to-Band Transitions, Phonon Modes, and Oxidation Properties of Cobalt-Oxide Core-Shell Slanted Columnar Thin Films. *Appl. Phys. Lett.* **2016**, *108* (5), 051905. https://doi.org/10.1063/1.4941399.

(21) Shirai, H.; Morioka, Y.; Nakagawa, I. Infrared and Raman Spectra and Lattice Vibrations of Some Oxide Spinels. *J. Phys. Soc. Jpn.* **1982**, *51* (2), 592–597. https://doi.org/10.1143/JPSJ.51.592.

(22) Meena, P. L.; Ravi Kumar; Sreenivas, K. Structural, Elastic and Magnetic Properties of Spinel Co_3O_4. *Indian J. Pure Appl. Phys. IJPAP* **2018**, *56* (11), 890–895.

(23) Kuzmenko, A. B. Kramers–Kronig Constrained Variational Analysis of Optical Spectra. *Rev. Sci. Instrum.* **2005**, *76* (8), 083108. https://doi.org/10.1063/1.1979470.

(24) Qiao, L.; Xiao, H. Y.; Meyer, H. M.; Sun, J. N.; Rouleau, C. M.; Puretzky, A. A.; Geohegan, D. B.; Ivanov, I. N.; Yoon, M.; Weber, W. J.; Biegalski, M. D. Nature of the Band Gap and Origin of the Electro-/Photo-Activity of Co_3O_4. *J. Mater. Chem. C* **2013**, *1* (31), 4628. https://doi.org/10.1039/c3tc30861h.

(25) Zviagin, V.; Sturm, C.; Esquinazi, P. D.; Grundmann, M.; Schmidt-Grund, R. Control of Magnetic Properties in Spinel $ZnFe_2O_4$ Thin Films through Intrinsic Defect Manipulation. *J. Appl. Phys.* **2020**, *128* (16), 165702. https://doi.org/10.1063/5.0019712.

(26) Wang, R.-P.; Huang, M.-J.; Hariki, A.; Okamoto, J.; Huang, H.-Y.; Singh, A.; Huang, D.-J.; Nagel, P.; Schuppler, S.; Haarman, T.; Liu, B.; de Groot, F. M. F. Analyzing the Local Electronic

Structure of Co_3O_4 Using 2p3d Resonant Inelastic X-Ray Scattering. *J. Phys. Chem. C* **2022**, *126* (20), 8752–8759. https://doi.org/10.1021/acs.jpcc.2c01521.

(27) Miedzinska, K. M. E.; Hollebone, B. R.; Cook, J. G. An Assigment of the Optical Absorption Spectrum of Mixed Valence Co_3O_4 Spinel Films. *J. Phys. Chem. Solids* **1987**, *48* (7), 649–656. https://doi.org/10.1016/0022-3697(87)90154-5.

(28) Callen, E. Optical Absorption Edge of Magnetic Semiconductors. *Phys. Rev. Lett.* **1968**, *20* (19), 1045–1048. https://doi.org/10.1103/PhysRevLett.20.1045.

(29) Roth, W. L. The Magnetic Structure of Co_3O_4. *J. Phys. Chem. Solids* **1964**, *25* (1), 1–10. https://doi.org/10.1016/0022-3697(64)90156-8.

(30) Campostrini, M.; Hasenbusch, M.; Pelissetto, A.; Rossi, P.; Vicari, E. Critical Exponents and Equation of State of the Three-Dimensional Heisenberg Universality Class. *Phys. Rev. B* **2002**, *65* (14), 144520. https://doi.org/10.1103/PhysRevB.65.144520.

(31) Zaharko, O.; Cervellino, A.; Tsurkan, V.; Christensen, N. B.; Loidl, A. Evolution of Magnetic States in Frustrated Diamond Lattice Antiferromagnetic $Co(Al_{1-x}Co_x)_2O_4$ Spinels. *Phys. Rev. B* **2010**, *81* (6), 064416. https://doi.org/10.1103/PhysRevB.81.064416.

(32) Campostrini, M.; Pelissetto, A.; Rossi, P.; Vicari, E. Improved High-Temperature Expansion and Critical Equation of State of Three-Dimensional Ising-like Systems. *Phys. Rev. E* **1999**, *60* (4), 3526–3563. https://doi.org/10.1103/PhysRevE.60.3526.

(33) Khriplovich, L. M.; Kholopov, E. V.; Paukov, I. E. Heat Capacity and Thermodynamic Properties of Co_3O_4 from 5 to 307 K Low-Temperature Transition. *J. Chem. Thermodyn.* **1982**, *14* (3), 207–217. https://doi.org/10.1016/0021-9614(82)90012-X.

(34) Waegele, M. M.; Doan, H. Q.; Cuk, T. Long-Lived Photoexcited Carrier Dynamics of d – d Excitations in Spinel Ordered Co_3O_4. *J. Phys. Chem. C* **2014**, *118* (7), 3426–3432. https://doi.org/10.1021/jp4113443.

(35) Jiang, C.-M.; Baker, L. R.; Lucas, J. M.; Vura-Weis, J.; Alivisatos, A. P.; Leone, S. R. Characterization of Photo-Induced Charge Transfer and Hot Carrier Relaxation Pathways in Spinel Cobalt Oxide (Co_3O_4). *J. Phys. Chem. C* **2014**, *118* (39), 22774–22784. https://doi.org/10.1021/jp5071133.

(36) Baldini, E.; Kubacka, T.; Mallett, B. P. P.; Ma, C.; Koohpayeh, S. M.; Zhu, Y.; Bernhard, C.; Johnson, S. L.; Carbone, F. Lattice-Mediated Magnetic Order Melting in $TbMnO_3$. *Phys. Rev. B* **2018**, *97* (12), 125149. https://doi.org/10.1103/PhysRevB.97.125149.

(37) Stevens, T. E.; Kuhl, J.; Merlin, R. Coherent Phonon Generation and the Two Stimulated Raman Tensors. *Phys. Rev. B* **2002**, *65* (14), 144304. https://doi.org/10.1103/PhysRevB.65.144304.

(38) Zeiger, H. J.; Vidal, J.; Cheng, T. K.; Ippen, E. P.; Dresselhaus, G.; Dresselhaus, M. S. Theory for Displacive Excitation of Coherent Phonons. *Phys. Rev. B* **1992**, *45* (2), 768–778. https://doi.org/10.1103/PhysRevB.45.768.

(39) Wdowik, U. D.; Parlinski, K. Lattice Dynamics of CoO from First Principles. *Phys. Rev. B* **2007**, *75* (10), 104306. https://doi.org/10.1103/PhysRevB.75.104306.

(40) Sakurai, J.; Buyers, W. J. L.; Cowley, R. A.; Dolling, G. Crystal Dynamics and Magnetic Excitations in Cobaltous Oxide. *Phys. Rev.* **1968**, *167* (2), 510–518. https://doi.org/10.1103/PhysRev.167.510.

(41) Gielisse, P. J.; Plendl, J. N.; Mansur, L. C.; Marshall, R.; Mitra, S. S.; Mykolajewycz, R.; Smakula, A. Infrared Properties of NiO and CoO and Their Mixed Crystals. *J. Appl. Phys.* **1965**, *36* (8), 2446–2450. https://doi.org/10.1063/1.1714508.

(42) Thomsen, C.; Strait, J.; Vardeny, Z.; Maris, H. J.; Tauc, J.; Hauser, J. J. Coherent Phonon Generation and Detection by Picosecond Light Pulses. *Phys. Rev. Lett.* **1984**, *53* (10), 989–992. https://doi.org/10.1103/PhysRevLett.53.989.

(43) Kappus, W. Lattice Dynamics of Sapphire (Corundum): Part II: Calculations of the Phonon Dispersion. *Z. Für Phys. B Condens. Matter Quanta* **1975**, *21* (4), 325–331. https://doi.org/10.1007/BF01325391.

(44) Doig, K. I.; Aguesse, F.; Axelsson, A. K.; Alford, N. M.; Nawaz, S.; Palkar, V. R.; Jones, S. P. P.; Johnson, R. D.; Synowicki, R. A.; Lloyd-Hughes, J. Coherent Magnon and Acoustic Phonon Dynamics in Tetragonal and Rare-Earth-Doped $BiFeO_3$ Multiferroic Thin Films. *Phys. Rev. B* **2013**, *88* (9), 094425. https://doi.org/10.1103/PhysRevB.88.094425.

(45) Sun, Y.; Meng, F.; Lee, C.; Soll, A.; Zhang, H.; Ramesh, R.; Yao, J.; Sofer, Z.; Orenstein, J. Dipolar Spin Wave Packet Transport in a van Der Waals Antiferromagnet. *Nat. Phys.* **2024**, *20* (5), 794–800. https://doi.org/10.1038/s41567-024-02387-2.

(46) Bossini, D.; Dal Conte, S.; Terschanski, M.; Springholz, G.; Bonanni, A.; Deltenre, K.; Anders, F.; Uhrig, G. S.; Cerullo, G.; Cinchetti, M. Femtosecond Phononic Coupling to Both Spins and Charges in a Room-Temperature Antiferromagnetic Semiconductor. *Phys. Rev. B* **2021**, *104* (22), 224424. https://doi.org/10.1103/PhysRevB.104.224424.

(47) Zhang, J.; Lian, Q.; Pan, Z.; Bai, W.; Yang, J.; Zhang, Y.; Tang, X.; Chu, J. Spin-phonon Coupling and Two-magnons Scattering Behaviors in Hexagonal NiAs-type Antiferromagnetic MnTe Epitaxial Films. *J. Raman Spectrosc.* **2020**, *51* (8), 1383–1389. https://doi.org/10.1002/jrs.5928.

(48) Chubukov, A. V.; Frenkel, D. M. Resonant Two-Magnon Raman Scattering in Antiferromagnetic Insulators. *Phys. Rev. Lett.* **1995**, *74* (15), 3057–3060. https://doi.org/10.1103/PhysRevLett.74.3057.

(49) Sugai, S.; Suzuki, H.; Takayanagi, Y.; Hosokawa, T.; Hayamizu, N. Carrier-Density-Dependent Momentum Shift of the Coherent Peak and the LO Phonon Mode in *p*-Type High-T_c Superconductors. *Phys. Rev. B* **2003**, *68* (18), 184504. https://doi.org/10.1103/PhysRevB.68.184504.

(50) Mijiti, Y.; Chen, K.; Rodrigues, J. E. F. S.; Hu, Z.; Nataf, L.; Trapananti, A.; Di Cicco, A.; Baudelet, F. Crystal and Electronic Structure of Co_3O_4 Spinel under Pressure Probed by XANES and Raman Spectroscopy. *Phys. Rev. B* **2021**, *103* (2), 024105. https://doi.org/10.1103/PhysRevB.103.024105.
